# The interferon-stimulated gene product oligoadenylate synthetase-like protein enhances replication of Kaposi’s sarcoma-associated herpesvirus (KSHV) and interacts with the KSHV ORF20 protein

**DOI:** 10.1371/journal.ppat.1006937

**Published:** 2018-03-02

**Authors:** Kendra A. Bussey, Ulrike Lau, Sophie Schumann, Antonio Gallo, Lisa Osbelt, Markus Stempel, Christine Arnold, Josef Wissing, Hans Henrik Gad, Rune Hartmann, Wolfram Brune, Lothar Jänsch, Adrian Whitehouse, Melanie M. Brinkmann

**Affiliations:** 1 Viral Immune Modulation Research Group, Helmholtz Centre for Infection Research (HZI), Braunschweig, Germany; 2 School of Molecular and Cellular Biology and Astbury Centre for Structural Molecular Biology, Faculty of Biological Sciences, University of Leeds, Leeds, United Kingdom; 3 Heinrich Pette Institute, Leibniz Institute for Experimental Virology, Hamburg, Germany; 4 Cellular Proteomics Research Group, Helmholtz Centre for Infection Research (HZI), Braunschweig, Germany; 5 Center for Structural Biology, Department of Molecular Biology and Genetics, Aarhus University, Aarhus, Denmark; 6 Institute of Virology, Hannover Medical School, Hannover, Germany; University of Pennsylvania Medical School, UNITED STATES

## Abstract

Kaposi’s sarcoma-associated herpesvirus (KSHV) is one of the few oncogenic human viruses known to date. Its large genome encodes more than 85 proteins and includes both unique viral proteins as well as proteins conserved amongst herpesviruses. KSHV ORF20 is a member of the herpesviral core UL24 family, but the function of ORF20 and its role in the viral life cycle is not well understood. ORF20 encodes three largely uncharacterized isoforms, which we found were localized predominantly in the nuclei and nucleoli. Quantitative affinity purification coupled to mass spectrometry (q-AP-MS) identified numerous specific interacting partners of ORF20, including ribosomal proteins and the interferon-stimulated gene product (ISG) oligoadenylate synthetase-like protein (OASL). Both endogenous and transiently transfected OASL co-immunoprecipitated with ORF20, and this interaction was conserved among all ORF20 isoforms and multiple ORF20 homologs of the UL24 family in other herpesviruses. Characterization of OASL interacting partners by q-AP-MS identified a very similar interactome to that of ORF20. Both ORF20 and OASL copurified with 40S and 60S ribosomal subunits, and when they were co-expressed, they associated with polysomes. Although ORF20 did not have a global effect on translation, ORF20 enhanced RIG-I induced expression of endogenous OASL in an IRF3-dependent but IFNAR-independent manner. OASL has been characterized as an ISG with antiviral activity against some viruses, but its role for gammaherpesviruses was unknown. We show that OASL and ORF20 mRNA expression were induced early after reactivation of latently infected HuARLT-rKSHV.219 cells. Intriguingly, we found that OASL enhanced infection of KSHV. During infection with a KSHV ORF20stop mutant, however, OASL-dependent enhancement of infectivity was lost. Our data have characterized the interaction of ORF20 with OASL and suggest ORF20 usurps the function of OASL to benefit KSHV infection.

## Author summary

The herpesviruses are a family of large double-stranded DNA viruses that cause a variety of illnesses from chicken pox to cancer. Kaposi’s sarcoma-associated herpesvirus (KSHV) is a cancer-causing herpesvirus and can lead to development of Kaposi’s sarcoma, a major form of cancer in HIV-positive patients. As for all herpesviruses, infection with KSHV is lifelong. Exactly how KSHV initiates and maintains its infection is still not well understood, but it must manipulate the host cell to establish favorable conditions. Likewise, the host has developed a complicated system to fight off invaders, which includes the production of interferon-stimulated gene products. We have now found that KSHV exploits one such host cell protein, the oligoadenylate synthetase-like protein (OASL). Rather than OASL acting as an antiviral protein as it does during many other viral infections, KSHV appears to have found a way to utilize OASL for its own benefit. The KSHV protein ORF20 interacts with OASL, they co-localize in nucleoli, and both ORF20 and OASL associate and purify with components of the cellular translational machinery. This may help viral infection by selectively controlling protein production.

## Introduction

The oncogenic human herpesvirus 8, also known as Kaposi’s sarcoma-associated herpesvirus (KSHV), belongs to the *Gammaherpesvirinae* subfamily of the *Herpesviridae* [1]. KSHV is the etiological agent of multiple malignancies, including Kaposi’s sarcoma, which is a common form of cancer in HIV-infected individuals [2], and primary effusion lymphoma, an aggressive non-Hodgkin’s lymphoma [3]. After successful primary infection of the host, KSHV, like all herpesviruses, can undergo lytic replication or establish latency in infected cells [4]. While the mechanisms leading to transformation and oncogenesis are still not completely understood, the current view is that both lytic replication and latency are important for KS tumor development [4].

KSHV has a large dsDNA genome with more than 85 open reading frames (ORFs) encoding proteins at least 100 amino acids (aa) in length. In addition, it utilizes mechanisms like splicing, mRNA editing, and alternative start codons to further maximize genomic capacity [5]. The poorly characterized ORF20 is an example of this additional complexity. Wildtype ORF20 (ORF20WT) encodes 3 colinear isoforms: the 320 amino acid long full length isoform ORF20FL, the 297 amino acid isoform ORF20A, and the 257 amino acid isoform ORF20B. The sequences for ORF20FL (AAC57101.1) and ORF20B (ABD28871.1), both of which start with a methionine, were submitted to Genbank as part of the genomic annotations for the minor and predominant (M and P) strains of KSHV, respectively, despite the presence of the sequences encoding ORF20FL and ORF20B in both genomes. ORF20A was identified much more recently and its translation utilizes a noncanonical leucine via a CTG start codon [5].

ORF20 is a member of the herpesviral core UL24 gene family and is conserved in the *Alpha-*, *Beta-*, and *Gammaherpesvirinae*. Transiently expressed HSV-1 UL24, KSHV ORF20WT, HCMV UL76, and MHV68 ORF20 have been reported to affect the mitotic cdc2/cyclinB complex, thereby inducing cell cycle arrest and apoptosis [6, 7]. However, studies with MHV68 WT and ORF20 mutant viruses found no effect on the cell cycle [8]. Multiple UL24 family members, including HSV-1 UL24, HCMV UL76, and KSHV ORF20WT, localize to the nucleoli of transfected cells [9, 10]. KSHV ORF20 mRNA is expressed late upon *de novo* infection of primary human umbilical vein endothelial cells. ORF20 mRNA is also detected upon reactivation of a latently infected body cavity based lymphoma cell line (BCBL-1) [11]. To date, the function of ORF20 is not well understood, and furthermore, the biological relevance of the different ORF20 isoforms is unclear.

Interferon-stimulated gene (ISG) transcription, including that of 2’-5’-oligoadenylate synthetase-like protein (OASL), is activated upon pattern recognition receptor and type I interferon (IFN) receptor signaling [12–15]. ISGs have evolved as a host defense mechanism and fall into three different categories: broadly acting antiviral effectors, ISGs with targeted antiviral specificity, and proviral ISGs that enhance the replication of certain viruses [15]. OASL expression is induced by viral infection. For example, both Sendai virus and influenza virus infection upregulate OASL in an early IFN regulatory factor 3 (IRF3)-dependent manner [13]. In a screen of almost 400 ISGs that characterized their antiviral activity against six different viruses, OASL was identified as an ISG with targeted antiviral specificity [15].

Lentiviral expression of OASL inhibited replication of hepatitis C virus, but not replication of human immunodeficiency virus type-1, yellow fever virus, West Nile virus, chikungunya virus, or Venezuelan equine encephalitis virus [15]. In a subsequent study that also primarily focused on RNA viruses, OASL inhibited poliovirus, equine arterivirus, and Newcastle disease virus, mildly inhibited influenza A virus and measles virus, but did not inhibit coxsackie B virus, Sindbis virus AR86, Sindbis virus Girdwood, o’nyong-nyong virus, human parainfluenza virus type 3, respiratory syncytial virus, bunyamwera virus, nor did it inhibit the DNA virus vaccinia [16]. The role of OASL for HSV-1 is more controversial; in one study, OASL expression was shown to have no effect on HSV-1 replication [17], and in another, OASL inhibited HSV-1 [18].

OASL is a member of the oligoadenylate synthetase protein family that includes the prominent enzymes cyclic GMP-AMP (cGAMP) synthase (cGAS) and 2’-5’-oligoadenylate synthase 1 (OAS1), as well as OAS2 and OAS3. The OAS enzymes and cGAS are activated by cytosolic double-stranded nucleic acids and produce 2’-5’-linked second messenger molecules [19]. While OASL shares a highly conserved N-terminal OAS-like domain with the OAS enzymes, it lacks enzymatic activity and has a unique C-terminus composed of two ubiquitin-like domains (UBL) [20]. Additionally, OASL has a double-stranded RNA (dsRNA) binding groove and the OAS-like domain binds dsRNA [21].

OASL is conserved between mice and humans; murine OASL1 (mOASL1) is the most similar to human OASL (hOASL), and like hOASL, mOASL1 lacks enzymatic activity and has a UBL [22]. mOASL2 is more distantly related to hOASL; it possesses enzymatic activity, unlike hOASL, but like OASL has a UBL [22]. mOASL1 has been shown to inhibit IRF7 translation by binding to the 5’ untranslated region (UTR) of IRF7, and compared to wildtype mice, mOASL1 knockout mice are more resistant to HSV-1 and EMCV infection, likely due to increased interferon production [23]. However, unlike mOASL, hOASL does not seem to bind to the IRF7 UTR [18]. A yeast two-hybrid screen that utilized a human leukocyte cDNA library identified the transcriptional repressor methyl CpG binding protein 1 (MBD1) as an interacting partner of OASL [24]. Another study found that hOASL enhanced RIG-I signaling, and furthermore showed that OASL co-immunoprecipitated with RIG-I [18]. To date, however, the function of OASL is not completely understood and no viral binding partners have previously been identified. Furthermore, its role during gammaherpesvirus infections is unknown.

We have now characterized all three isoforms of KSHV ORF20 and show that they localize to the nuclei and nucleoli of transiently transfected or transduced cells, including HeLa, 293T, primary HFF, HuARLT2, and HuARLT2-rKSHV.219. In an unbiased quantitative affinity purification coupled to mass spectrometry (q-AP-MS) approach, we identify OASL as an interaction partner of ORF20. We show that the interaction with OASL is conserved among ORF20 isoforms and UL24 family members present in other herpesviruses. We analyze the OASL interactome by q-AP-MS and find that OASL and ORF20 share numerous ribosomal interaction partners. Furthermore, both proteins copurify with 40S and 60S ribosomal subunits. Moreover, when expressed together they associate with polysomes, but do not have a global effect on translation. Interestingly, ORF20 upregulates OASL mRNA expression downstream of RIG-I in an IRF3-dependent manner, but independently of IFNAR signaling. During reactivation of latently infected HuARLT2-rKSHV.219, both OASL and ORF20 mRNA levels are upregulated early. Lastly, OASL enhances KSHV infection in an ORF20-dependent manner, suggesting that KSHV has commandeered this ISG to benefit KSHV infection.

## Results

### KSHV ORF20 encodes three isoforms with predominantly nuclear and nucleolar localization

**Fig 1:**
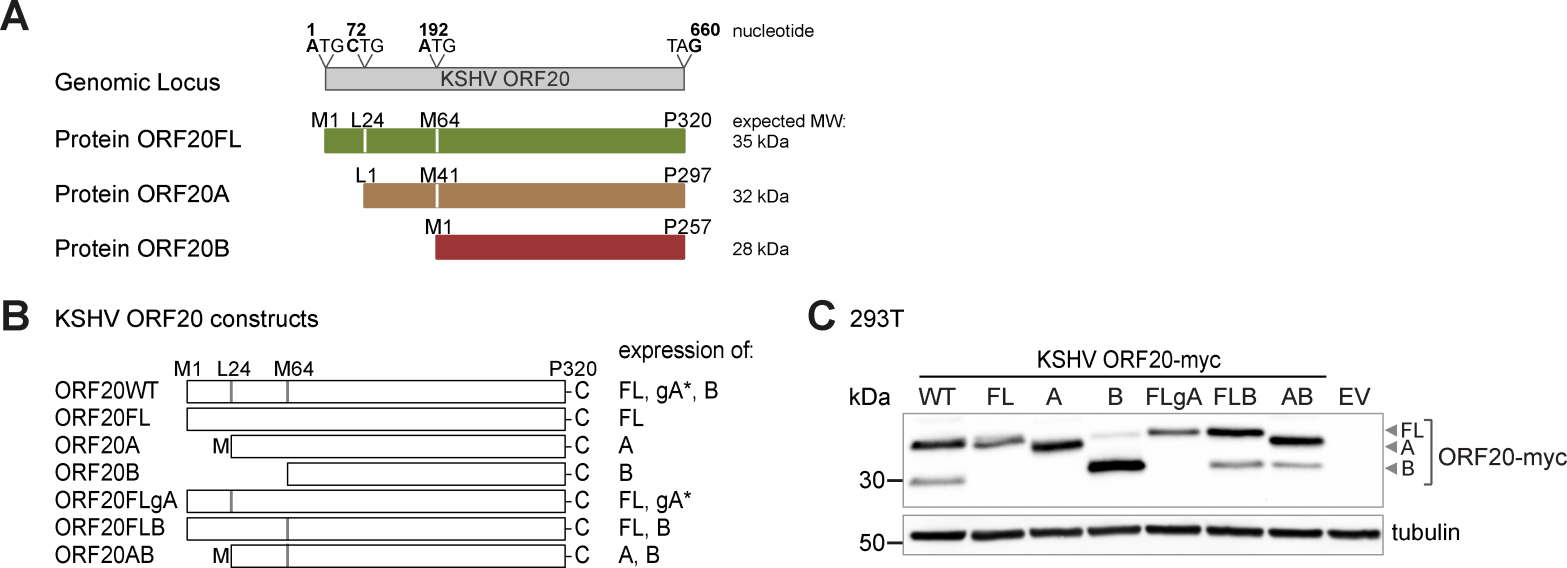
KSHV ORF20 encodes three isoforms. (A) KSHV ORF20WT, a member of the UL24 family, can potentially express three isoforms: ORF20FL, starting at methionine 1 (M1) (aa 1–320), ORF20A, starting at leucine 24 (L24) (aa 1–297), and ORF20B, starting at M64 (aa 1–257). (B) Plasmid constructs express the three isoforms singly or in combination with each other as indicated. For analysis of ORF20A, either the genomic L24 start codon (ORF20WT and ORF20FLgA) or genomic L24 with an upstream methionine, indicated with M (ORF20A, ORF20AB) was used. * indicates genomic ORF20A leucine start codon; ORF20A starting with leucine was not detectable by immunoblotting. (C) Expression vectors encoding ORF20WT, individual ORF20 isoforms, or ORF20 isoforms in combination with each other were transfected into 293T cells. Lysates were prepared 24 h later, separated by Bis-Tris PAGE, and anti-myc and anti-tubulin immunoblotting was performed. Empty vector (EV) was included as a control. The immunoblot is representative of four independent experiments.

KSHV ORF20 is a member of the conserved UL24 family but to date it has not been extensively characterized. The ORF20 genomic locus encoded on the minus strand of the genome is 660 nucleotides in length and although the 5’ and 3’ untranslated regions are not known [5], it is anticipated that one mRNA is transcribed (Fig 1A). Next generation ribosomal footprinting data identified initiating ribosomes at three positions, giving rise to three colinear isoforms of ORF20: full-length ORF20 (ORF20FL) as well as two shorter isoforms, ORF20A, which starts at an alternative leucine start codon at amino acid 24 of ORF20FL, and ORF20B, which starts at an internal methionine at amino acid 64 of ORF20FL (Fig 1A) [5]. The anticipated sizes of all three protein isoforms are 35, 32, and 28 kDa, respectively (Fig 1A). A plasmid construct encoding genomic ORF20WT [10], equipped with a C-terminal myc epitope tag, can potentially express ORF20FL, ORF20A, and ORF20B (Fig 1B), but we only observed two distinct bands upon immunoblotting cell lysates with an anti-myc antibody (Fig 1C). To identify the isoforms responsible for these two bands, we cloned all isoforms singly and in combination (Fig 1B) and analyzed their expression by immunoblotting (Fig 1C). We found that the two bands observed upon expression of ORF20WT correspond to ORF20FL and ORF20B (Fig 1C). We could not detect expression of ORF20A from ORF20WT or ORF20FLgA plasmid constructs that contain the genomic leucine start codon, but could drive expression of ORF20A by addition of an N-terminal methionine in ORF20A and ORF20AB constructs (Fig 1B and 1C).

**Fig 2:**
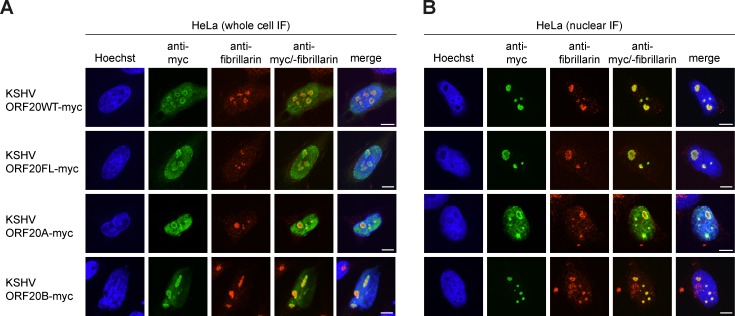
KSHV ORF20 isoforms localize predominantly to the nuclei and nucleoli. (A-B) HeLa cells were transfected with the indicated plasmid and seeded onto coverslips. 48 h post transfection, (A) coverslips were fixed directly in 4% paraformaldehyde in PBS (PFA) for whole cell immunofluorescence. (B) For nuclear immunofluorescence, coverslips were incubated in cold 1% NP-40 extraction buffer for 5 min to remove the cytoplasm before PFA fixation. (A-B) All samples were then processed for anti-myc (green) and anti-fibrillarin (red) immunofluorescence. Nuclei were counterstained with Hoechst (blue). Images are representative of three independent experiments. Scale bar = 10 μm.

A previous study has shown that KSHV ORF20WT localizes to the nucleus [10]. However, the localization of all individual ORF20 isoforms has not yet been studied. We determined the subcellular localization of ORF20WT as well as the individual ORF20 isoforms ORF20FL, ORF20A, and ORF20B in HeLa cells by immunofluorescence (IF) and confocal microscopy. First, we analyzed the localization of ORF20 isoforms in whole cell IF. All isoforms localized predominantly to the nuclei and nucleoli, where they co-localized with Hoechst and the nucleolar protein fibrillarin, but in some cells ORF20 forms could also be detected in the cytoplasm (Fig 2A). To enhance visualization of the nucleoli, we performed nuclear IF. For this, we extracted the cytoplasm by incubation with 1% NP-40 extraction buffer prior to fixation. By labeling the nucleolar marker fibrillarin, we confirmed that all KSHV ORF20 isoforms localized to the nucleoli (Fig 2B).

### ORF20 interacts with ribosomal proteins and the oligoadenylate synthetase-like protein OASL

**Fig 3:**
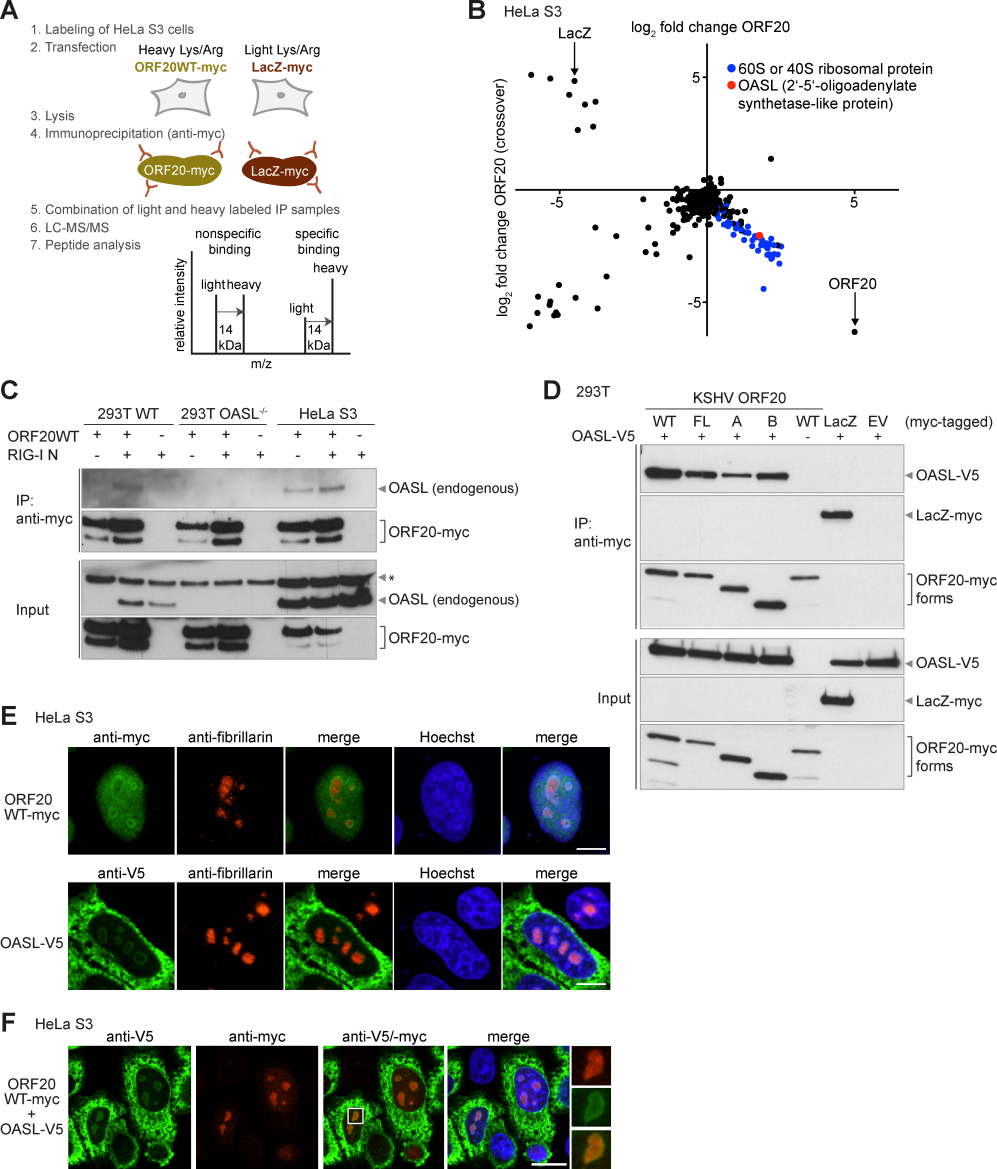
Quantitative affinity purification coupled to mass spectrometry identifies OASL as an ORF20 interaction partner. (A) Scheme of the quantitative affinity purification coupled to mass spectrometry (q-AP-MS) workflow used to identify cellular interaction partners of ORF20. In the forward experiment, HeLa S3 cells were labeled with heavy or light amino acids, then transfected with ORF20WT-myc or LacZ-myc, respectively. In the crossover experiment, ORF20WT-myc was labeled with light amino acids and LacZ-myc with heavy amino acids. Lysates were immunoprecipitated with anti-myc magnetic beads, combined for affinity purification, processed, and analyzed by liquid chromatography-tandem mass spectrometry (LC-MS/MS). ORF20 interaction partners were identified based on the increased abundance of heavy labeled proteins compared to light labeled proteins. In the parallel crossover experiment, specific interaction partners were identified based on increased abundance of light labeled proteins. Proteins with a 1:1 ratio of light and heavy amino acids are non-specific binding partners or contaminants. (B) Quantitative proteomics results displayed graphically. The abundances of proteins identified in the forward and crossover experiments are graphed, with ORF20 interacting partners located in the lower right quadrant. Each point represents one protein. 40S and 60S ribosomal proteins are shown in blue and OASL is indicated in red. (C) 293T, 293T OASL^-/-^, and HeLa S3 cells were transfected with ORF20WT-myc (+) or empty vector (-) and/or RIG-I N (+) or empty vector (-). An anti-myc immunoprecipitation of NP-40 lysates was performed and input lysates and immunoprecipitates were immunoblotted with anti-OASL and anti-myc antibodies. *: Nonspecific background band. Immunoblots are representative of two independent experiments. (D) 293T cells were transfected with myc-tagged ORF20 isoforms, LacZ-myc, OASL-V5, and/or EV as indicated. An anti-myc immunoprecipitation of RIPA lysates was performed and input lysates and immunoprecipitates were immunoblotted with anti-V5 and anti-myc antibodies. Data are representative of three independent experiments. (E) HeLa S3 cells were transfected individually with ORF20WT-myc or OASL-V5, then labeled with antibodies against myc or V5 as appropriate (green) and fibrillarin (red). (F) HeLa S3 cells were co-transfected with ORF20WT-myc and OASL-V5, and then labeled with anti-V5 (green) and anti-myc (red) antibodies. Nuclei were counterstained with Hoechst (blue). Images are representative of three independent experiments. Scale bar = 10 μm (E) and 20 μm (F).

To better understand the function of ORF20, we utilized q-AP-MS [25, 26] to identify cellular binding partners of ORF20. HeLa S3 cells were metabolically labeled with stable heavy or light isotopes of arginine and lysine, then transfected with myc-tagged ORF20WT or LacZ as a control. In the forward experiment, heavy labeled cells were transfected with ORF20-myc, and light labeled cells with LacZ-myc (Fig 3A). In the crossover experiment, the labels were exchanged. Lysates were subjected to anti-myc immunoprecipitation, then combined and subjected to LC/MS-MS before peptide analysis (Fig 3A). In the forward experiment, specific interaction partners of ORF20 were more abundant in the heavy form than in the light form (Fig 3A), while in the crossover experiment the label switch resulted in an inverted abundance of the same protein. In contrast, nonspecific binding partners were equally abundant in the forward and crossover experiment. Proteins identified in both the forward and crossover experiments were analyzed for relative ratios and graphed. Interacting partners of LacZ are displayed in the upper left quadrant, and nonspecific interactions or contaminants are located around the origin (Fig 3B). We identified multiple specific interacting partners of ORF20 (Fig 3B, Table 1, and S1 Dataset), which cluster in the lower right quadrant of the graph (Fig 3B). These interacting partners included numerous 40S and 60S ribosomal proteins, ribosome-binding protein 1, and OASL, an interferon-stimulated gene product (ISG) (Table 1, S1 Dataset, S1 Supporting Information). The 40S and 60S ribosomal subunits, which contain many individual proteins as well as the 5.8S, 18S, and 28S ribosomal RNAs (rRNAs), form the mature 80S eukaryotic ribosome. Multiple 80S ribosomes associate to form actively translating ribosomes or polysomes [27, 28].

**Table 1:** ORF20 interacting partners identified by q-AP-MS analysis.

Protein	Log_2_ forward	Log_2_ crossover
60S ribosomal protein L36	2,510	-2,515
40S ribosomal protein S12	2,452	-3,281
60S ribosomal protein L10a	2,396	-2,876
Ribosome-binding protein 1	2,391	-2,467
60S ribosomal protein L15	2,272	-2,858
40S ribosomal protein S24	2,270	-2,461
60S ribosomal protein L24	2,200	-3,036
40S ribosomal protein S6	2,178	-3,063
60S acidic ribosomal protein P1	2,173	-2,173
60S ribosomal protein L7a	2,126	-2,422
60S ribosomal protein L4	2,111	-2,867
60S acidic ribosomal protein P0	2,049	-2,598
60S ribosomal protein L34	2,044	-2,889
60S ribosomal protein L8	2,027	-2,385
60S ribosomal protein L27	2,009	-2,400
60S ribosomal protein L6	2,002	-2,516
60S ribosomal protein L18	1,926	-2,353
60S ribosomal protein L30	1,913	-4,404
60S acidic ribosomal protein P2	1,860	-2,224
60S ribosomal protein L35a	1,851	-2,386
60S ribosomal protein L3	1,831	-2,463
**2'-5'-oligoadenylate synthase-like protein (OASL)**	**1,774**	**-2,035**
40S ribosomal protein S26	1,731	-2,700
60S ribosomal protein L7	1,712	-2,114
60S ribosomal protein L12	1,625	-2,055
60S ribosomal protein L23	1,542	-2,238
60S ribosomal protein L10	1,530	-2,498
40S ribosomal protein S2	1,404	-1,914
40S ribosomal protein S23	1,383	-1,631
60S ribosomal protein L19	1,293	-1,886
BAG family molecular chaperone regulator 2	1,284	-0,940
60S ribosomal protein L14	1,254	-2,225
28S ribosomal protein S28	1,165	-1,810
60S ribosomal protein L13	1,157	-1,756
Heterogeneous nuclear ribonucleoprotein F	1,150	-1,401
Protein LTV1 homolog	1,150	-1,512
U4/U6 small nuclear ribonucleoprotein Prp3	1,034	-0,998
60S ribosomal protein L29	1,005	-1,531
40S ribosomal protein S14	0,898	-1,993
40S ribosomal protein S3a	0,981	-1,722
40S ribosomal protein S8	0,990	-1,678
40S ribosomal protein S4, X isoform	0,713	-1,617
60s ribosomal protein L27a	0,784	-1,513
Apoptosis-inducing factor 1, mitochondrial	0,878	-1,498
60S ribosomal protein L17	0,714	-1,432
U4/U6 small nuclear ribonucleoprotein Prp31	0,776	-1,222
Pre-mRNA-processing factor 6	0,776	-1,051

Highly confident interaction partners were based on log_2_ fold change values with an absolute value ≥1 in one experiment and ≥ 0.7 in the other experiment. The complete list can be found in S1 Dataset.

We next wanted to confirm the interaction of ORF20 with endogenous OASL in wildtype 293T and HeLa S3 cells, using 293T OASL^-/-^ cells as a specificity control (Fig 3C). As OASL is an ISG, we transfected cells with a constitutively active mutant of the pattern recognition receptor RIG-I, RIG-I N, to enhance expression of endogenous OASL. Endogenous OASL was specifically expressed in RIG-I N transfected 293T cells, but not in the absence of RIG-I N transfection or in 293T OASL^-/-^ cells. In HeLa S3 cells, OASL was detected both in the presence and absence of RIG-I N. We immunoprecipitated ORF20WT with an anti-myc antibody, then analyzed immunoprecipitates for the presence of endogenous OASL by anti-OASL immunoblotting. OASL co-immunoprecipitated with ORF20WT from both 293T and HeLa S3 cells co-transfected with RIG-I N, but not from 293T OASL^-/-^ cells, as expected. OASL also co-immunoprecipitated with ORF20WT from HeLa S3 cells in the absence of RIG-I N, validating our q-AP-MS results (Fig 3C, Table 1).

As detection of endogenous OASL was challenging, we used co-transfections for further interaction studies. To determine whether OASL interacted with all ORF20 isoforms, we co-transfected 293T cells with V5-tagged OASL and various ORF20-myc isoform constructs or LacZ-myc as a control. All proteins were appropriately expressed in the input lysates (Fig 3D). We then performed an anti-myc immunoprecipitation and immunoblotted first against V5-OASL, then verified successful immunoprecipitation of the various myc constructs (Fig 3D). We found that OASL co-immunoprecipitated with all ORF20 isoforms (FL, A, and B) individually as well as with ORF20WT, but not with the LacZ control.

We then analyzed the subcellular localization of OASL and ORF20 by immunofluorescence. We tested several antibodies for detection of endogenous OASL and could detect endogenous protein by immunoblotting but not by immunofluorescence. Furthermore, there are no commercial antibodies available against ORF20. Thus, to analyze localization, we transfected HeLa S3 cells with either myc-tagged ORF20WT or V5-tagged OASL, or co-transfected them with both plasmids. When expressed alone, ORF20WT was located predominantly in the nuclei and nucleoli (Fig 3E). When OASL was expressed alone, it was located in the cytoplasm as well as in the nucleoli (Fig 3E). When ORF20WT and OASL were co-expressed, their subcellular localization was unaltered (Fig 3F). ORF20WT and OASL co-localized in the nucleoli but not elsewhere in the cells (Fig 3F).

**Fig 4:**
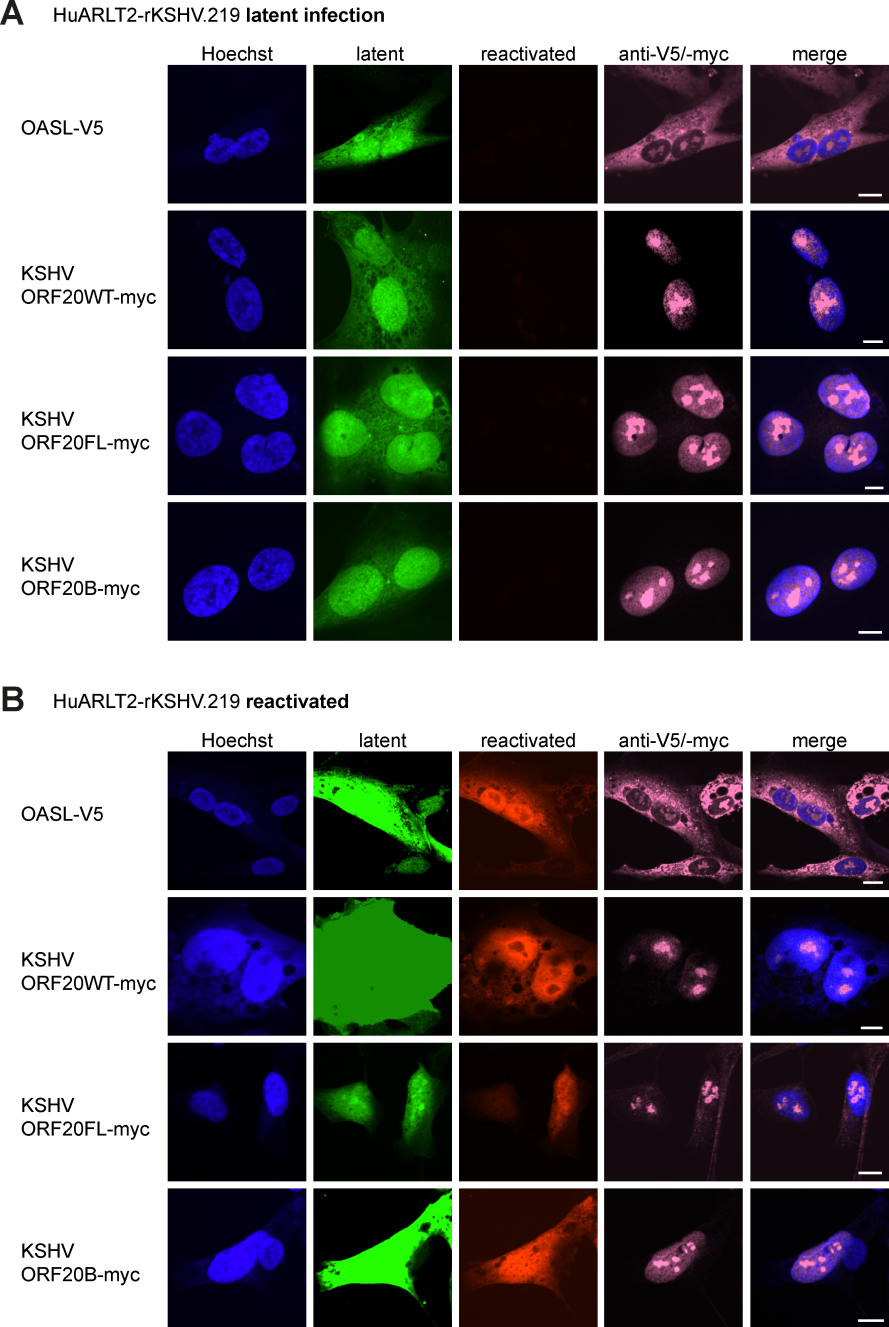
The subcellular localization of OASL-V5 and ORF20-myc isoforms is unaltered in latent and reactivated HuARLT2-rKSHV.219 cells. HuARLT2-rKSHV.219 cells were transiently transduced with lentiviruses encoding the indicated construct. 4 days post transduction, cells were seeded onto glass coverslips. The following day, cells were untreated (A, latent infection) or reactivated with sodium butyrate and RTA-expressing baculovirus (B, reactivated). Infected cells express GFP, reactivated cells express RFP, and transduced proteins were detected with anti-myc or anti-V5 antibodies and an anti-mouse Alexa Fluor 647 coupled antibody (pink). Nuclei were counterstained with Hoechst (blue). Images are representative of at least 2 independent experiments. Scale bar = 10 μm.

Next, we wanted to verify the subcellular localization of OASL and ORF20 in additional cell types. First, we utilized HuARLT2-rKSHV.219 cells, a conditionally immortalized human endothelial cell line latently infected with recombinant KSHV rKSHV.219 [29–32]. In cells infected with rKSHV.219, latently infected cells express GFP from the cellular EF-1α promoter and upon reactivation RFP from the KSHV lytic gene PAN promoter. We transduced HuARLT2-rKSHV.219 cells with lentiviruses encoding OASL-V5, ORF20WT-myc, ORF20FL-myc, or ORF20B myc, then analyzed the localization in latently infected cells (Fig 4A). We found that the localization was similar to that observed in HeLa cells; OASL was present in the cytoplasm and nucleoli, and ORF20 forms were present in the nuclei and nucleoli (Fig 4A). To determine whether the localization remained the same in reactivated cells, we reactivated transduced HuARLT2-rKSHV.219 cells with sodium butyrate and a baculovirus carrying the RTA gene (Fig 4B). As in latently infected cells, in reactivated cells, OASL was present in the cytoplasm and nucleoli, and ORF20 forms were identified in the nuclei and nucleoli (Fig 4B). We then analyzed the subcellular localization of OASL and ORF20 forms in transiently transduced primary human foreskin fibroblasts (S1A Fig) and conditionally immortalized HuARLT2 endothelial cells (S1B Fig). The subcellular localization was similar to that observed in other cell types; OASL was detected in the cytoplasm and nucleoli, and ORF20 forms in the nuclei and nucleoli.

Next, to determine whether the localization of ORF20 was dependent upon OASL expression, we transfected 293T or 293T OASL^-/-^ cells with ORF20WT and analyzed the localization by immunofluorescence. In both cell types, ORF20WT was detected in the nuclei and nucleoli (S1C Fig). In parallel, we co-transfected 293T cells with ORF20WT and RIG-I N to induce expression of endogenous OASL. Due to lack of specific antibodies for IF of endogenous OASL, we verified nuclear translocation of endogenous IRF3 as a marker for OASL expression. In ORF20WT expressing cells with nuclear translocation of IRF3, ORF20WT localized to the nuclei and nucleoli (S1D Fig), similar to what we saw in HeLa cells, where endogenous OASL was detected even in the absence of RIG-I N (Fig 3C).

In summary, ORF20 forms were consistently detected in the nuclei and nucleoli in primary and immortalized cells and the subcellular localization of ORF20 was independent of OASL expression. OASL was observed in the cytoplasm and nucleoli in a variety of cell types, and its localization was independent of ORF20 expression, and ORF20 and OASL co-localized in the nucleoli of transiently transfected cells.

As ORF20 and OASL co-localized in the nucleoli, we wanted to verify whether their interaction detected by IP was specific or only due to their subcellular co-localization. To do so, we cloned an ORF20WT-myc-GFP fusion and compared it to GFP-NS1A, in which GFP is fused to amino acids 203–237 of influenza A/Udorn/72 NS1, conferring nucleolar localization of GFP [33]. As a positive control, we utilized GFP-tagged RIG-I, which localizes to the cytoplasm, as RIG-I has previously been shown to interact with OASL under mild lysis conditions [18]. We co-transfected OASL-V5 with two independent ORF20WT-myc-GFP clones, RIG-I-GFP, or GFP-NS1A, as well as all singly-transfected controls, and performed an anti-V5 IP (S2A Fig). We found that ORF20-GFP co-immunoprecipitated with OASL-V5, but GFP-NS1A did not. RIG-I-GFP also weakly co-immunoprecipitated with OASL-V5. Next, we performed an anti-GFP IP on similar samples and found that OASL-V5 strongly co-immunoprecipitated with ORF20-myc-GFP, but not with GFP-NS1A (S2B Fig). Under our stringent lysis conditions, OASL-V5 did not co-immunoprecipitate with RIG-I-GFP (S2B Fig). These data confirm the interaction of ORF20 with OASL and verify that the detected interaction is not solely due to nucleolar co-localization.

### The interaction with OASL is conserved among UL24 family members

**Fig 5:**
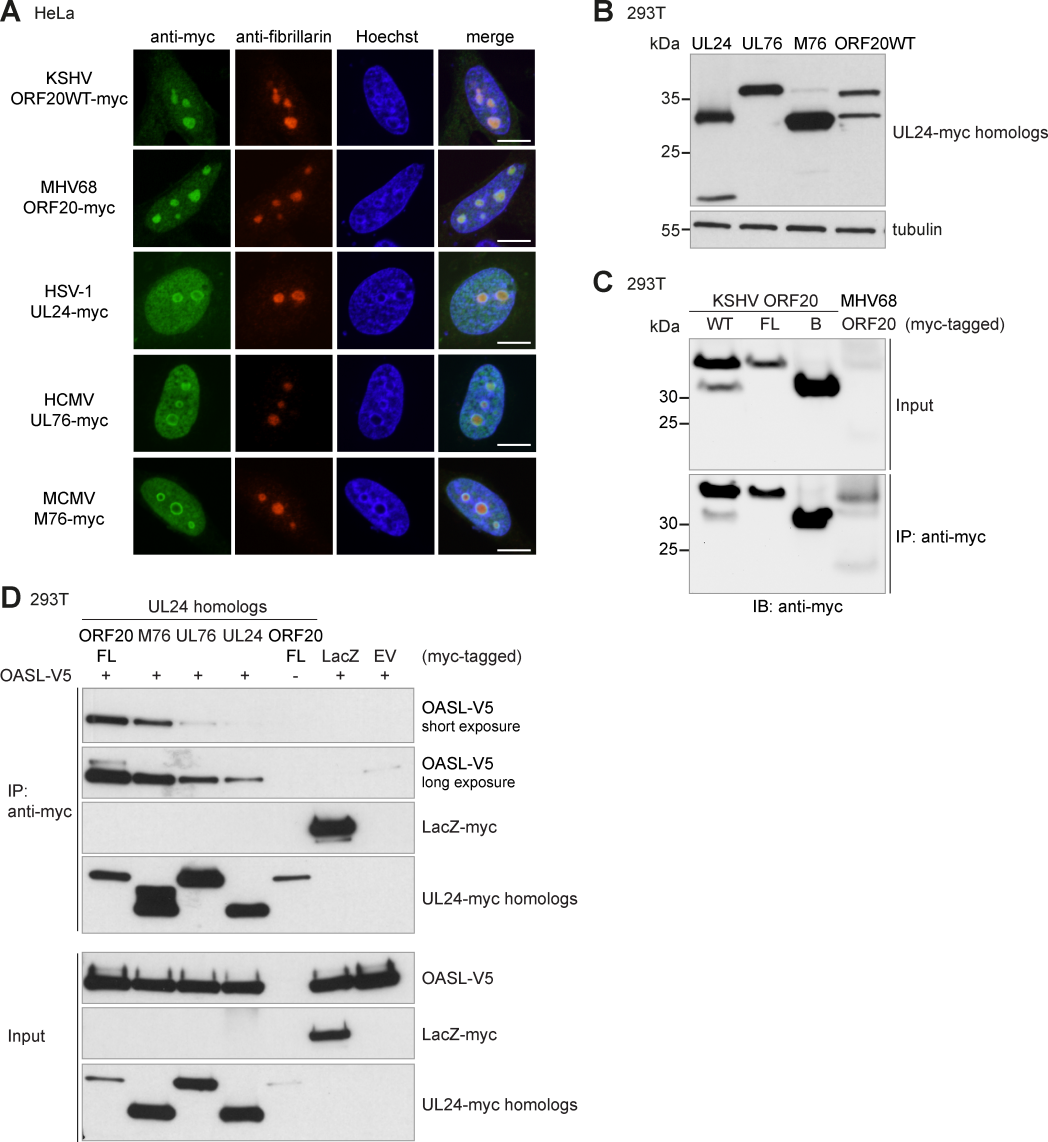
The interaction of ORF20 with OASL is conserved among the members of the UL24 family. (A) HeLa cells were transfected with the indicated plasmid and seeded onto coverslips. 48 h post transfection, coverslips were fixed in PFA and processed for anti-myc (green) and anti-fibrillarin (red) immunofluorescence. Nuclei were counterstained with Hoechst (blue). Images are representative of three independent experiments. Scale bar = 10 μm (B) 293T cells were transfected with myc-tagged UL24, UL76, M76, or ORF20WT. Lysates were prepared 24 h later, separated by SDS-PAGE, and anti-myc and anti-tubulin immunoblotting was performed. Data are representative of four independent experiments. (C) 293T cells were transfected with the indicated myc-tagged KSHV ORF20 form or MHV68 ORF20. An anti-myc immunoprecipitation of RIPA lysates was performed and input lysates and immunoprecipitates were immunoblotted with an anti-myc antibody. Data are representative of two independent experiments. (D) 293T cells were transfected with myc-tagged UL24 homologs MCMV M76, HCMV UL76, or HSV-1 UL24, LacZ-myc, OASL-V5, and/or EV as indicated. An anti-myc immunoprecipitation of RIPA lysates was performed and input lysates and immunoprecipitates were immunoblotted with anti-V5 and anti-myc antibodies. Data are representative of two independent experiments.

We next wanted to determine if the interaction with OASL was specific for KSHV ORF20 or if it was conserved among UL24 family members present in the *Alpha-*, *Beta-*, and *Gammaherpesvirinae*. We compared the amino acid sequences of the UL24 family members HSV-1 UL24, HCMV UL76, MCMV M76, KSHV ORF20FL, KSHV ORF20A, KSHV ORF20B, and MHV68 ORF20, and found that all KSHV ORF20 homologs aligned to each other and all forms of ORF20 (S3 Fig). We cloned myc-tagged constructs utilizing the genomic nucleotide sequence of several KSHV ORF20 homologs: HSV-1 UL24, HCMV UL76, and MCMV M76. As MHV68 ORF20 was poorly expressed, we codon-optimized its sequence and added a 3x myc tag. First, we analyzed the subcellular localization of all homologs in transiently transfected HeLa cells. Similarly to ORF20, all UL24 family members were located in the nuclei and nucleoli (Fig 5A). We verified expression of all homologs by immunoblotting, and found that UL24, M76, and ORF20B were of similar sizes, while UL76 and MHV68 ORF20 were more similar to the size of ORF20WT (Fig 5B and 5C). While codon-optimized MHV68 ORF20 expression was low compared to KSHV ORF20, it was expressed and detection of MHV68 ORF20 was improved by anti-myc immunoprecipitation prior to immunoblotting (Fig 5C).

To determine whether OASL interacted with UL24 family members, we co-transfected 293T cells with V5-tagged OASL and myc-tagged KSHV ORF20FL, MCMV M76, HCMV UL76, or HSV-1 UL24, or LacZ-myc as a control. OASL co-immunoprecipitated strongly with KSHV ORF20FL and MCMV M76. HCMV UL76 and HSV-1 UL24 also immunoprecipitated OASL, but much more weakly than KSHV ORF20 (Fig 5D). OASL-V5 did not co-immunoprecipitate with the negative control LacZ (Fig 5D). Our results show that the interaction with OASL is conserved among ORF20 isoforms and UL24 homologs.

### The interaction of ORF20 with OASL is independent of RNA binding by OASL

**Fig 6:**
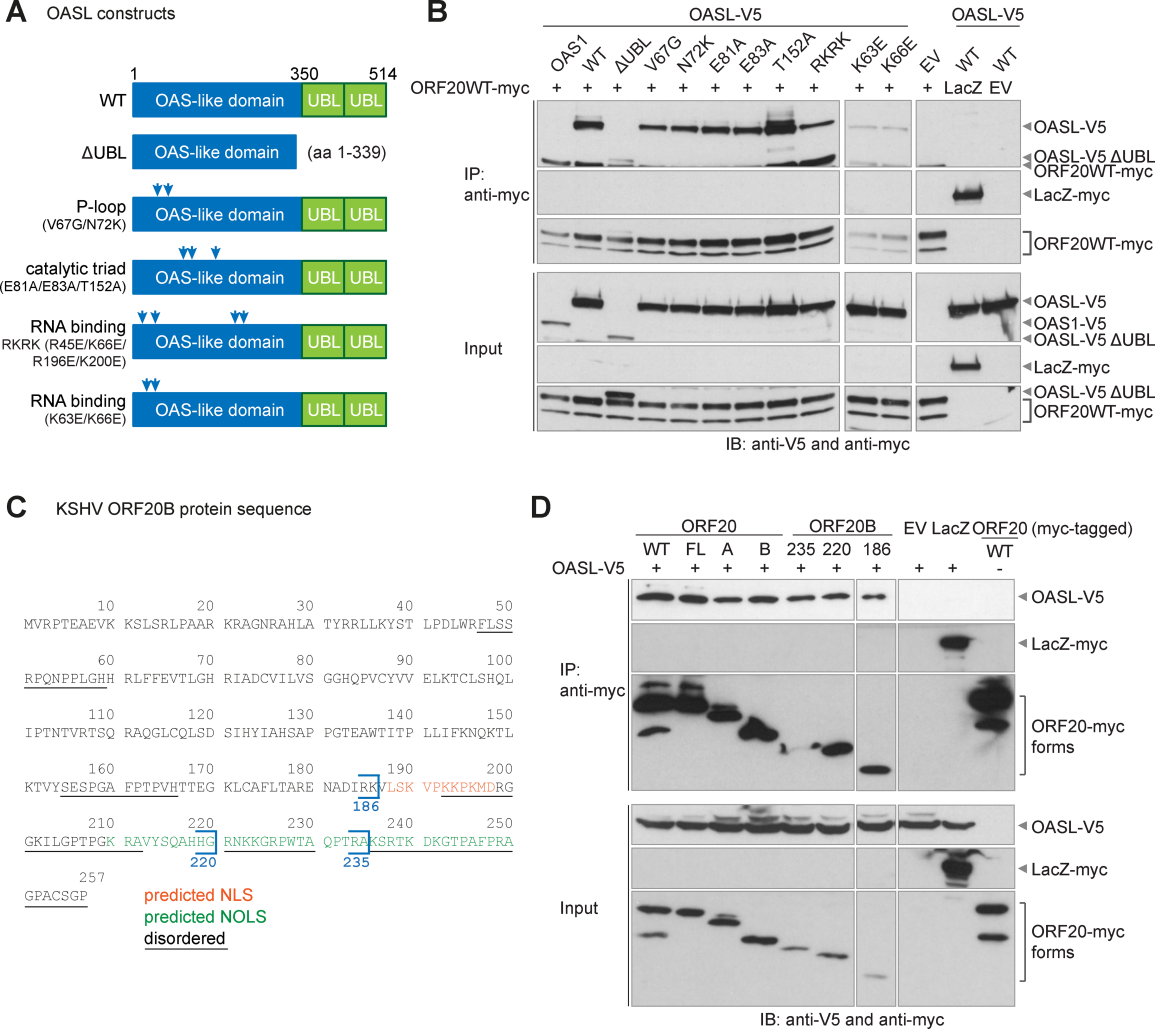
OASL RNA binding and ubiquitin-like domains are not required for interaction with ORF20. (A) Multiple OASL constructs were utilized, including WT OASL and OASL mutants, including ΔUBL lacking the ubiquitin-like domain, the P-loop mutants V67G and N72K, the RNA binding mutants R45E/K66E/R196E/K200E (RKRK), K63E, and K66E, and the catalytic triad mutants, E81A, E83A, and T152A. (B) 293T cells were transfected with myc-tagged ORF20WT or LacZ, V5-tagged OAS1, WT or mutant OASL, and/or empty vector (EV) as indicated. An anti-myc immunoprecipitation of RIPA lysates was performed and input lysates and immunoprecipitates were immunoblotted with anti-V5 and anti-myc antibodies. Immunoblots are representative of at least three independent experiments. Similar results were obtained with simultaneous and sequential antibody exposures. (C) The amino acid sequence for ORF20B is shown, with the predicted nuclear localization sequence indicated in red, the predicted nucleolar localization sequence indicated in green, and predicted disordered regions underlined. The predictions formed the basis for three ORF20B C-terminal truncation mutants: ORF20B 1–186, 1–220, and 1–235. (D) 293T cells were transfected with myc-tagged ORF20 isoforms, ORF20B mutants, or LacZ, OASL-V5, and/or EV as indicated. An anti-myc immunoprecipitation of RIPA lysates was performed. Input lysates and immunoprecipitates were immunoblotted with anti-V5 and anti-myc antibodies. Immunoblots are representative of three independent experiments.

We next analyzed the interaction of ORF20 with a variety of V5-tagged OASL mutants to determine if specific OASL domains or functions are required for the interaction to occur. The mutants included ΔUBL, lacking the ubiquitin-like domain of OASL; P-loop mutants V67G and N72K with reduced nucleic acid binding; three RNA binding mutants, R45E/K66E/R196E/K200E (RKRK), K63E, and K66E; and three catalytic triad mutants, E81A, E83A, and T152A, based on homology with the catalytic site of the OAS family enzymes [21] (Fig 6A). We also used OAS1, which is enzymatically-active and lacks the UBL domains, as a control in our interaction studies. We co-transfected myc-tagged ORF20WT with V5-tagged OAS1, WT OASL, or the various OASL mutants and verified their expression in input lysates (Fig 6B). All constructs were expressed, although OAS1 and OASLΔUBL were weakly expressed compared to WT OASL. We then performed an anti-myc immunoprecipitation and found that WT OASL and all OASL mutants co-immunoprecipitated with ORF20WT, but not with the negative control LacZ (Fig 6B). OASLΔUBL, despite its weaker expression compared to WT OASL, also co-immunoprecipitated with ORF20WT, but OAS1 did not, confirming the specificity of the interaction between ORF20 and OASL. We next verified the subcellular localization of all OASL constructs by whole-cell and nuclear immunofluorescence, and found that with the exception of OASLΔUBL, which was weakly expressed and detected in the cytoplasm and nuclei, all constructs were localized to the cytoplasm and nucleoli (S4 Fig).

As all three ORF20 isoforms immunoprecipitated OASL, we next wanted to identify the region of ORF20B required for interaction with OASL. Based on secondary structure and nuclear and nucleolar localization sequence predictions (Fig 6C), we created three ORF20B deletion mutants: ORF20B 1–235, 1–220, and 1–186 (Fig 6C). We co-transfected the myc-tagged ORF20 isoforms WT, FL, A, or B, ORF20B deletion mutants, or LacZ as a control with V5-tagged OASL. OASL and all ORF20 isoforms and truncation mutants were expressed in input lysates as expected (Fig 6D). We then performed an anti-myc immunoprecipitation and found that OASL co-immunoprecipitated with all ORF20B deletion mutants, suggesting that the first 186 amino acids of ORF20B are important for the interaction with OASL (Fig 6D). We then verified the subcellular localization of all ORF20B deletion mutants and found that all mutants localized to the nuclei and nucleoli (S5 Fig). These data suggest that the predicted nuclear and nucleolar localization sequences (Fig 6C) are not exclusively required for ORF20 localization. In summary, neither the ubiquitin-like domains nor RNA binding functions of OASL are required for the interaction with ORF20, and the interaction of ORF20 with OASL can be mapped to amino acids 1–186 of the smallest isoform, ORF20B.

### OASL and ORF20 share very similar interactomes

**Fig 7:**
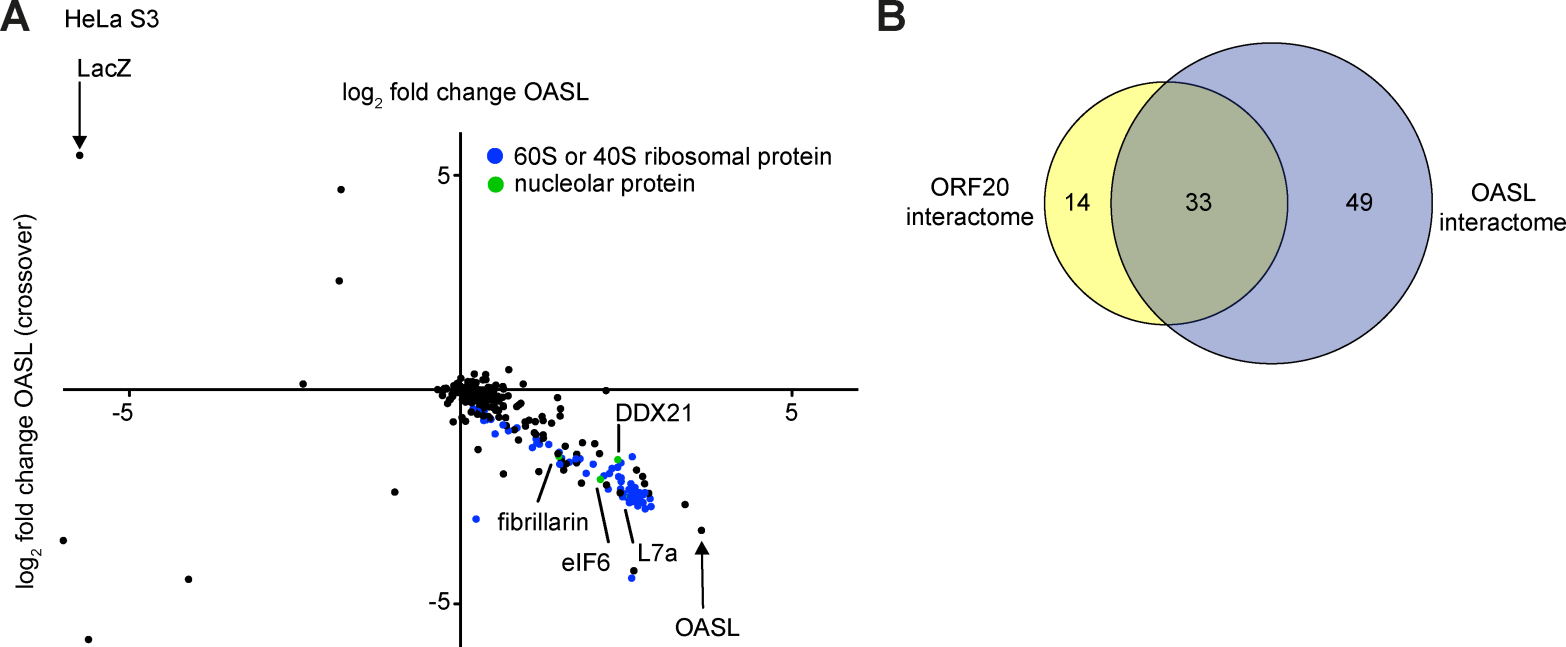
Quantitative proteomics identified nucleolar and ribosomal interaction partners of OASL. (A) q-AP-MS was performed as shown in Fig 3A. For the forward experiment, heavy-labeled HeLa S3 cells were transfected with OASL-myc and light-labeled cells were transfected with LacZ-myc. In the crossover experiment, OASL-myc was labeled with light amino acids and LacZ-myc with heavy amino acids. Cell lysates were immunoprecipitated with anti-myc magnetic beads and analyzed by LC-MS/MS. Specific interacting partners of OASL identified in both the forward and crossover experiments are located in the lower right quadrant. 40S and 60S ribosomal proteins are shown in blue and nucleolar proteins are indicated in green. (B) The interacting partners shown in Tables 1 and 2 were compared using VennDis to determine the number of specific and shared interacting partners for ORF20 and OASL (related to S1 Supporting Information).

To better understand why ORF20 and OASL may interact, we utilized unbiased q-AP-MS to identify interaction partners of OASL. Using an experimental setup similar to that for ORF20 (Fig 3A), we identified numerous interacting partners of OASL (Fig 7A, Table 2, S2 Dataset), of which many were 40S or 60S ribosomal proteins or nucleolar proteins. We also identified proteins with anticipated functions in ribosome biogenesis (Table 2, S2 Dataset). Next, we compared the proteins identified as interacting partners of ORF20 and OASL (Table 1 and Table 2). We identified 14 proteins that copurified with ORF20 only and 49 proteins that copurified exclusively with OASL. Interestingly, 33 proteins were identified as interacting partners for both ORF20 and OASL (Fig 7B and S1 Supporting Information).

**Table 2:** OASL interacting partners identified by q-AP-MS.

Protein	Log_2_ forward	Log_2_ crossover
Ribosome production factor 2 homolog	3,388	-2,682
60S ribosomal protein L36	2,875	-2,727
40S ribosomal protein S10	2,858	-2,546
RNA-binding protein 34	2,838	-2,419
60S ribosomal protein L5	2,784	-2,782
Ubiquitin-40S ribosomal protein S27a	2,782	-2,403
Probable rRNA-processing protein EBP2	2,776	-2,193
60S ribosomal protein L10a	2,754	-2,453
40S ribosomal protein S28	2,753	-2,635
RNA-binding protein 28	2,747	-2,026
60S acidic ribosomal protein P0	2,698	-2,403
40S ribosomal protein S3	2,696	-2,544
40S ribosomal protein S12	2,675	-2,592
40S ribosomal protein S18	2,673	-2,712
40S ribosomal protein S16	2,672	-2,546
60S acidic ribosomal protein P1	2,671	-2,498
60S ribosomal protein L12	2,664	-2,553
Putative ribosomal RNA methyltransferase NOP2	2,656	-1,874
40S ribosomal protein S25	2,642	-2,567
60S ribosomal protein L8	2,631	-2,285
Ribosome biogenesis protein BRX1 homolog	2,614	-4,226
60S ribosomal protein L18a	2,614	-2,606
60S ribosomal protein L21	2,604	-2,550
60S ribosomal protein L34	2,601	-2,464
60S ribosomal protein L7	2,592	-1,567
60S acidic ribosomal protein P2	2,591	-2,371
60S ribosomal protein L30	2,582	-2,449
40S ribosomal protein S5	2,582	-4,397
60S ribosomal protein L35a	2,571	-2,194
60S ribosomal protein L28	2,558	-2,627
40S ribosomal protein S20	2,546	-2,635
60S ribosomal protein L14	2,541	-2,329
60S ribosomal protein L6	2,505	-2,508
60S ribosomal protein L7a	2,457	-2,470
60S ribosomal protein L27	2,453	-2,423
60S ribosomal protein L32	2,444	-2,497
60S ribosomal protein L3	2,423	-2,058
40S ribosomal protein S17-like	2,422	-2,144
60S ribosomal protein L4	2,419	-1,704
60S ribosomal protein L11	2,415	-2,318
Uncharacterized protein C7orf50	2,408	-2,409
60S ribosomal protein L13a	2,384	-2,028
Nucleolar RNA helicase 2	2,371	-1,632
60S ribosomal protein L18	2,368	-1,811
60S ribosomal protein L15	2,285	-1,836
60S ribosomal protein L37a	2,244	-1,956
60S ribosomal protein L13	2,228	-2,319
Ribosome biogenesis regulatory protein homolog	2,202	-2,221
40S ribosomal protein S15	2,162	-2,013
Eukaryotic translation initiation factor 6	2,111	-2,093
Putative oxidoreductase GLYR1	2,096	-1,492
Ataxin-2-like protein	2,023	-1,259
Ribosomal L1 domain-containing protein 1	2,001	-1,737
60S ribosomal protein L29	1,895	-1,952
Coiled-coil domain-containing protein 137	1,838	-1,235
Nuclease-sensitive element-binding protein 1	1,823	-2,181
60S ribosomal protein L24	1,803	-1,612
40S ribosomal protein S2	1,772	-1,653
Fragile X mental retardation syndrome-related protein 1	1,754	-1,507
Protein LLP homolog	1,751	-1,708
60S ribosomal protein L10	1,741	-1,616
60S ribosomal protein L9	1,640	-1,692
Y-box-binding protein 3	1,593	-1,728
Exosome complex component RRP46	1,578	-1,319
Double-stranded RNA-binding protein Staufen homolog 2	1,554	-1,874
60S ribosomal protein L23	1,526	-1,600
Polyadenylate-binding protein 1	1,520	-1,670
Elongation factor 1-alpha 1	1,508	-0,443
RNA-binding protein 10	1,502	-0,615
40S ribosomal protein S14	1,497	-1,739
40S ribosomal protein S8	1,487	-1,458
rRNA 2'-O-methyltransferase fibrillarin	1,480	-1,555
M-phase phosphoprotein 6	1,468	-1,491
60S ribosomal protein L19	1,331	-1,279
Nucleolar protein 16	1,249	-1,133
ATP-dependent RNA helicase A	1,246	-1,054
40S ribosomal protein S23	1,190	-1,270
Centromere protein V	1,181	-1,913
Heat shock protein beta-1	1,171	-1,197
60S ribosomal protein L27a	1,150	-1,245
40S ribosomal protein S6	1,138	-1,150
Heterochromatin protein 1-binding protein 3	1,133	-1,042
Vimentin	1,112	-1,006
40S ribosomal protein S3a	1,082	-1,349

Highly confident interaction partners were based on log_2_ fold change values with an absolute value ≥1 in one experiment and ≥ 0.7 in the other experiment. The complete list can be found in S2 Dataset.

### ORF20 and OASL co-sediment with ribosomal subunits and polysomes

**Fig 8:**
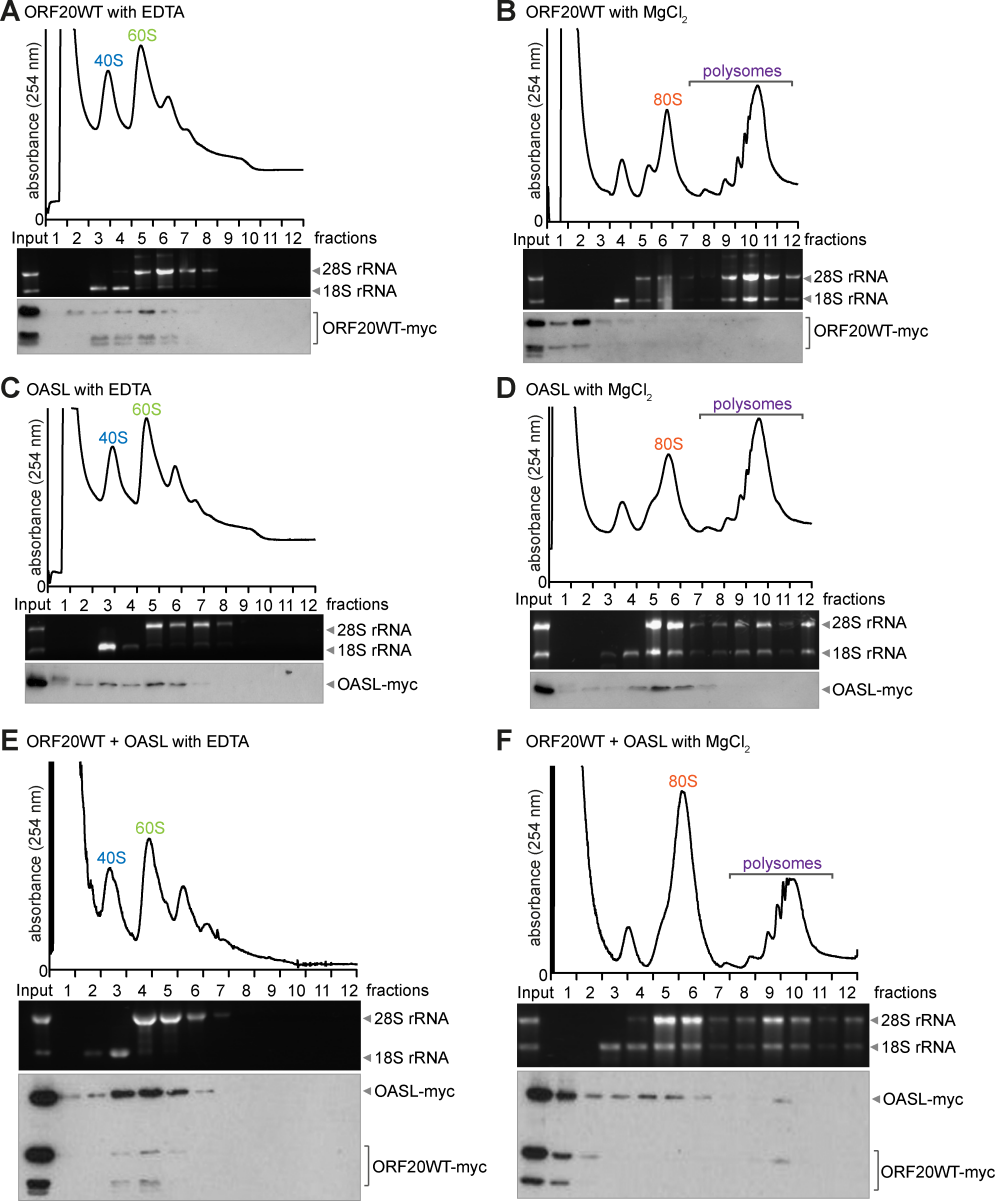
ORF20 and OASL copurify with ribosomal subunits and polysomes. 293T cells were cotransfected with myc-tagged ORF20 (A-B), OASL (C-D), or ORF20 and OASL together (E-F), then ribosomes were purified by 5–45% sucrose gradient fractionation in the presence of EDTA (A, C, E) or MgCl_2_ (B, D, F). Fractionation profiles were determined by absorbance at 254 nm. 40S and 60S indicate the small and large ribosomal subunits, respectively, and 80S indicates the monosome peak. Fractions were collected and analyzed for 18S and 28S rRNA by denaturing gel electrophoresis and for OASL and ORF20 expression by anti-myc immunoblotting. Results are representative of two independent experiments.

The nucleoli are the sites of ribosome biogenesis. Based on the nucleolar localization of ORF20 and OASL, as well as on their numerous shared nucleolar and ribosomal interacting partners, we wanted to verify whether ORF20 and OASL co-sediment with ribosomal subunits and/or polysomes. We performed sucrose gradient fractionation of ribosomes from cells expressing ORF20WT, OASL, or both ORF20WT and OASL together. Ribosomes were purified either in the presence of EDTA, which causes the dissociation of ribosomes into small (40S) and large (60S) subunits, or in the presence of MgCl_2_ to stabilize 80S ribosomes and polysomes. The absorbance at 254 nm was measured to identify the fractions containing subunits, ribosomes, and polysomes (Fig 8). The fraction identification was further verified by analyzing the 18S and 28S rRNA content; 18S rRNA was present in the 40S subunit fractions and 28S rRNA in the 60S subunit fractions. We found that when expressed alone, ORF20WT and OASL individually copurified with 40S and 60S ribosomal subunits (Fig 8A and 8C). ORF20WT weakly copurified with 80S ribosomes and polysomes (Fig 8B) and OASL copurified with 80S ribosomes when expressed alone (Fig 8D).

When co-expressed, ORF20WT and OASL copurified with 40S and 60S ribosomal subunits (Fig 8E), as well as with polysomes (Fig 8F). In summary, the co-sedimentation of ORF20WT and OASL with the 40S and 60S ribosomal subunits verifies our identification of 40S and 60S ribosomal subunit proteins as specific interaction partners of ORF20WT and OASL. Furthermore, the association of ORF20WT and OASL with polysomes suggests that these proteins may affect protein translation.

### ORF20 and OASL do not globally affect translation

**Fig 9:**
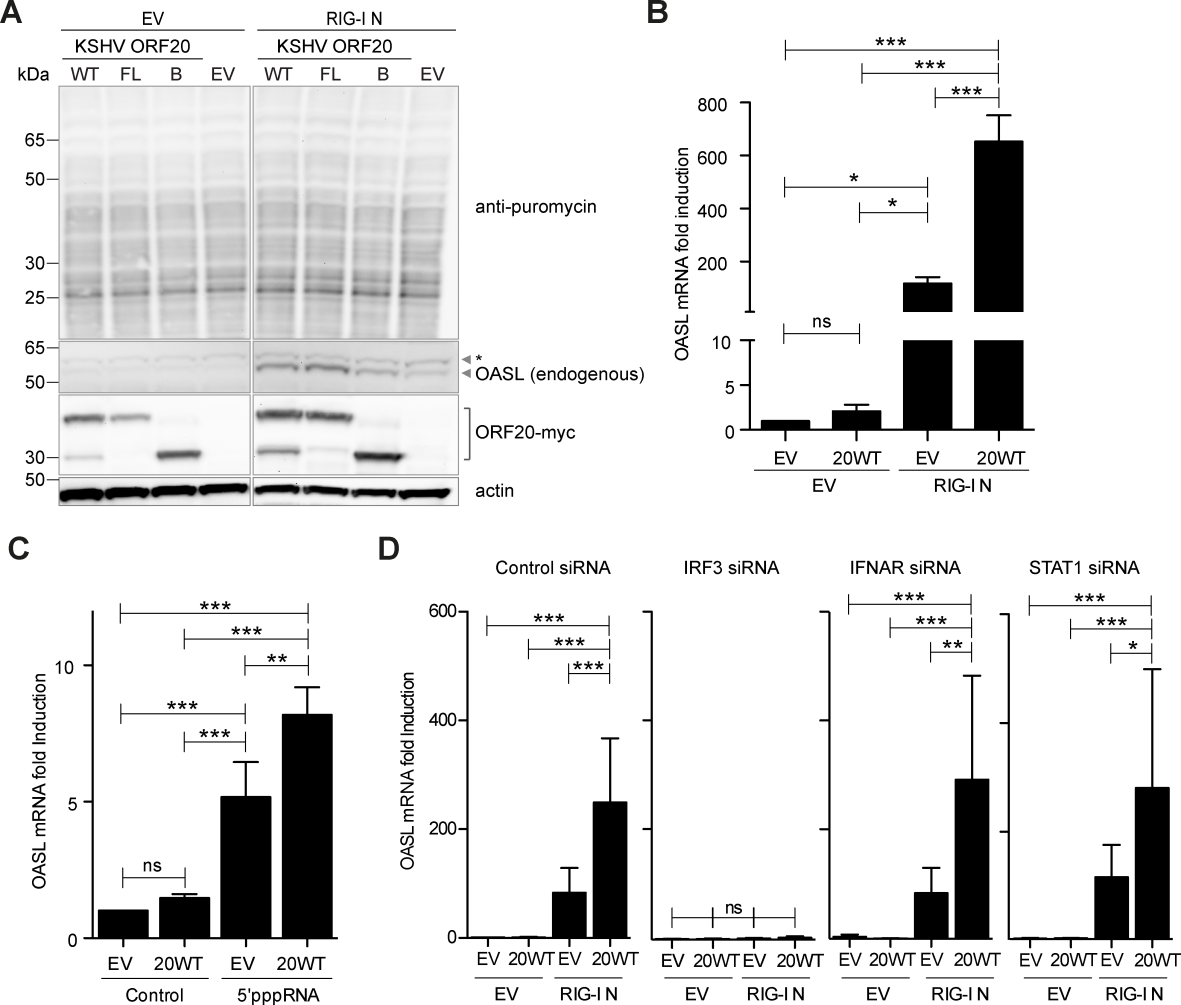
ORF20 does not affect translation rates, but enhances expression of endogenous OASL. (A) 293T cells were co-transfected with EV, myc-tagged ORF20WT, ORF20FL, or ORF20B, and either EV or RIG-I N. 24 h post transfection, cells were treated for 15 minutes with 5μg/ml puromycin, then lysed and analyzed by immunoblotting. Anti-puromycin, anti-OASL, anti-myc, and anti-actin immunoblotting was performed sequentially. Data are representative of two independent experiments. *: Nonspecific background band. (B) 293T cells were co-transfected with EV or ORF20WT-myc, and either EV or RIG-I N, for 24h. (C) HEK293 cells were transfected with either EV or ORF20WT-myc. 24h later, cells were transfected with 5’pppRNA complexed with Lipofectamine 2000 for approximately 24h. (D) 293T cells were reverse transfected with control, IRF3, IFNAR, or STAT1 siRNAs as indicated. 48h later, cells were transfected with EV or ORF20WT-myc, and either EV or RIG-I N, for 24h. (B, C, D) Cells were lysed, RNA was isolated, mRNA-specific cDNA was reverse transcribed, and the amount of OASL was determined using relative quantification to GAPDH levels and the 2^-ΔΔ*C*T^ method. Data shown are means + SD of combined duplicates from at least two experiments. ns, not significant, * P<0.05, ** P<0.01, *** P<0.001.

To analyze whether ORF20WT and OASL have global effects on cellular translation, we utilized a puromycin incorporation assay [34]. 293T cells were transfected with EV, ORF20WT, ORF20FL, or ORF20B, and either EV as a control or RIG-I N to induce expression of endogenous OASL. 24 h post transfection, cells were treated for 15 minutes with puromycin to allow incorporation into nascent proteins, then immediately lysed in sample buffer. Lysates were then subjected to anti-puromycin, anti-OASL, anti-myc, and anti-actin immunoblotting. Expression of ORF20 did not affect translation rates, as incorporation of puromycin was similar across all samples both in the absence and presence of RIG-I N (Fig 9A). As expected, we did not detect endogenous OASL unless RIG-I N was transfected. In addition, we did not observe global changes in translation upon OASL expression, as translation rates were similar in EV and RIG-I N transfected cells (Fig 9A). Interestingly, the amount of OASL protein was slightly increased when ORF20 forms were present (Fig 9A).

### ORF20 enhances OASL transcription

To determine whether ORF20 affects OASL mRNA expression, we used quantitative reverse transcriptase polymerase chain reaction (q-RT-PCR) to measure OASL mRNA levels in transfected 293T cells. As expected, OASL mRNA levels were upregulated in the presence of RIG-I N (Fig 9B). In addition, we observed a further significant increase in OASL mRNA levels when ORF20WT and RIG-I N were expressed (Fig 9B). As specificity controls, we included additional nuclear KSHV ORFs in this assay. Unlike for ORF20WT, compared to EV with RIG-I N, we observed no upregulation of OASL mRNA levels in cells co-expressing RIG-I N and either ORF59, K8.1, ORF27, or RTA (S6A Fig). We also included vIRF1 as a known inhibitor of IRF3 [35], and observed reduced OASL levels as expected (S6A Fig). Next, we determined whether ORF20 also enhanced OASL expression when endogenous RIG-I was stimulated. For this, we utilized HEK293 cells with functional RIG-I signaling, transfected them with empty vector or ORF20WT, and stimulated the cells with 5’pppRNA to activate the RIG-I signaling cascade (Fig 9C). We found that OASL mRNA levels were significantly upregulated upon 5’pppRNA stimulation, and as with RIG-I N (Fig 9B), OASL mRNA levels were significantly increased in the presence of ORF20WT (Fig 9C).

As OASL expression can be induced directly by IRF3 as well as via IFNAR signaling [13], we wanted to elucidate the mechanism required for enhanced expression of OASL in the presence of ORF20. To do so, we used siRNA to knockdown IRF3, IFNAR, or STAT1 expression, then transfected cells with combinations of empty vector, ORF20WT, and/or RIG-I N. In control siRNA-transfected cells, we observed upregulation of OASL in the presence of ORF20WT and RIG-I N (Fig 9D), as shown previously (Fig 9B). In cells transfected with siRNA targeting IRF3, IRF3 was efficiently knocked down (S6B Fig) and upregulation of OASL was completely abrogated (Fig 9D). Next, we analyzed the effect of IFNAR and STAT1 knockdown on the ability of ORF20WT to upregulate OASL mRNA levels and found that neither IFNAR nor STAT1 knockdown affected upregulation of OASL mRNA levels (Fig 9D), although expression of both was efficiently inhibited (S6C and S6D Fig, respectively).

In summary, these results suggest that although ORF20 does not globally affect translation, it affects expression of endogenous OASL at both the mRNA and protein levels, and ORF20-mediated upregulation of OASL mRNA expression is IRF3- but not IFNAR-dependent.

### ORF20 does not affect the interaction between OASL and RIG-I

As OASL has been shown to interact and co-localize with RIG-I [18], we next analyzed whether ORF20 affected the interaction or co-localization of OASL with RIG-I. First, we analyzed interactions in transiently transfected 293T cells lysed under milder conditions than those used for S2 Fig. OASL co-immunoprecipitated with RIG-I in both the presence and absence of ORF20WT (S7A Fig). ORF20WT did not interact with RIG-I in the absence of OASL, but did co-immunoprecipitate with RIG-I in the presence of OASL, suggesting the detected interaction was due to OASL binding both RIG-I and ORF20WT (S7A Fig).

Our analysis of the subcellular localization of RIG-I, OASL, and ORF20WT in HeLa S3 cells showed that RIG-I was located in the cytoplasm, as expected, OASL was in the cytoplasm and nucleoli, and ORF20WT in the nuclei and nucleoli (S7B Fig) as shown previously (Fig 3E and 3F, Fig 4, S1 Fig). When RIG-I and OASL were co-expressed, their subcellular localization did not change, and OASL and RIG-I co-localized in the cytoplasm (S7B Fig) as reported [18]. Co-expression of ORF20WT and RIG-I did not alter their subcellular localization, nor did they co-localize (S7B Fig). When RIG-I, OASL, and ORF20WT were co-expressed, neither their individual subcellular localization, nor the co-localization between ORF20WT and OASL in the nucleoli, nor the co-localization between RIG-I and OASL in the cytoplasm, was affected (S7C Fig). Co-localization between ORF20WT and RIG-I was also not observed in the presence of OASL (S7C Fig).

### OASL is proviral for gammaherpesviral infection

**Fig 10:**
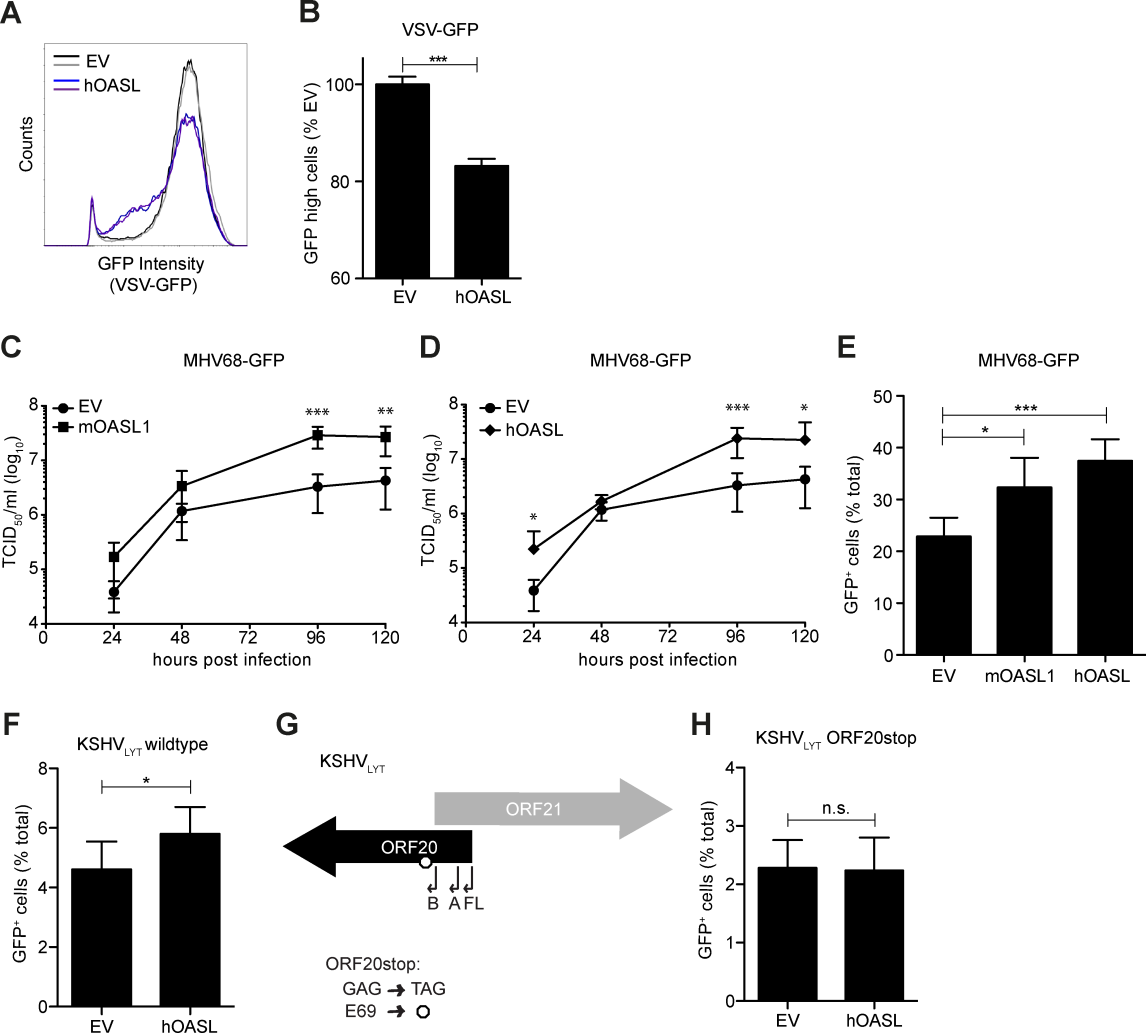
OASL expression is beneficial for MHV68 and KSHV replication. (A-F, H) 293T OASL^-/-^ cells were reconstituted with empty vector (EV), mOASL1, or hOASL as indicated. (A-B) 24 h later, cells were infected with VSV-GFP for 16 h. The GFP intensity in fixed cells was determined by flow cytometry. (A) Histograms of the VSV-GFP signal for duplicates from one representative experiment are shown. (B) The number of highly-GFP positive cells was determined and scaled to EV. Means + SD of duplicates from two independent experiments are shown. (C-D) 24 h post transfection, cells were infected with MHV68-GFP at an MOI of 0.05 for 2 h at 37°C. Supernatant was harvested at the indicated times and the viral titer was determined by TCID_50_ on M2-10B4 cells. Means ± SD of triplicates from two independent experiments are shown. (E, F, H) 24 h post transfection, cells were infected with (E) MHV68-GFP, (F) KSHV_LYT_ wildtype, or (H) KSHV_LYT_ ORF20stop. 20–24 h later, cells were detached, fixed, and the number of GFP-positive cells was determined by flow cytometry. Means + SD of 4–6 replicates total from two independent experiments are shown. (G) In KSHV_LYT_ ORF20stop, the ORF20 GAG codon for E69 is replaced with the stop codon TAG. For all subfigures: ns, not significant, * P<0.05, **P<0.01, *** P<0.001.

ISGs can have pro- and anti-viral effects [15]. Although OASL has been identified as an antiviral protein against multiple viruses, it has targeted antiviral specificity [15, 16]. To verify the antiviral function of OASL in a reconstitution assay, we infected 293T OASL^-/-^ cells reconstituted with EV or hOASL with vesicular stomatitis virus expressing GFP (VSV-GFP) (Fig 10A and 10B) and determined the GFP signal by flow cytometry 16 h post infection. Compared to the mean fluorescence intensity (MFI) of GFP in EV-transfected cells, we observed a decrease in MFI when hOASL was expressed (Fig 10A). We observed a corresponding significant decrease in the number of GFP-high cells when hOASL was expressed (Fig 10B), confirming the antiviral effect of OASL during VSV infection [18].

OASL has not been characterized for its role during gammaherpesviral infection. We therefore determined the effect of OASL expression on gammaherpesviral growth and infectivity. First, we reconstituted 293T OASL^-/-^ cells with the murine homolog of OASL, mOASL1 (Fig 10C), human OASL (Fig 10D), or empty vector (EV) as a control. We then infected cells with MHV68-GFP at a low multiplicity of infection (MOI) and performed growth curves over several days. When either mOASL1 or hOASL was expressed, viral growth was enhanced compared to cells transfected with EV (Fig 10C and 10D). We then reconstituted 293T OASL^-/-^ cells with mOASL1 or hOASL, infected them with MHV68-GFP at a high MOI, and quantified the number of infected GFP-positive cells by flow cytometry 20 h post infection. We found that the number of GFP-positive cells was significantly increased in samples expressing mOASL1 or hOASL, compared to cells transfected with EV (Fig 10E).

We next performed a similar experiment with KSHV. As KSHV infection *in vitro* frequently defaults to latency and ORF20 is a lytic gene, we used a genetically modified form of KSHV. In KSHV_LYT_, the viral replication and transcription activator RTA is under control of a constitutively active PGK promoter, leading to exclusively lytic replication [36, 37]. We infected 293T OASL^-/-^ cells reconstituted with either EV or hOASL with wildtype KSHV_LYT_ and used flow cytometry to quantify the number of infected cells 24 h post infection. We found that the number of GFP-positive cells was significantly increased in the presence of hOASL (Fig 10F). Our data show that OASL is beneficial for both MHV68 and KSHV_LYT_ infection.

To determine whether the proviral effect of OASL is dependent on the presence of ORF20, we used *en passant* mutagenesis to generate an ORF20stop virus on the KSHV_LYT_ background. To avoid affecting the ORF21 protein sequence or identified ORF21 promoter transcription factor binding sites, we changed the codon for E69, GAG, to the stop codon TAG (Fig 10G). While the first 69 amino acids of ORF20FL may be expressed, only 5 amino acids of ORF20B can be expressed. We infected 293T OASL^-/-^ cells reconstituted with either EV or hOASL with KSHV_LYT_ ORF20stop. We found that the number of GFP-positive cells was similar in both EV and hOASL reconstituted cells (Fig 10H), in contrast to the enhancement of infection observed for KSHV_LYT_ wildtype (Fig 10F). These data suggest that ORF20 contributes to the beneficial effect of hOASL on KSHV_LYT_.

### OASL and ORF20 mRNA are expressed in reactivated HuARLT2-rKSHV.219 cells

**Fig 11:**
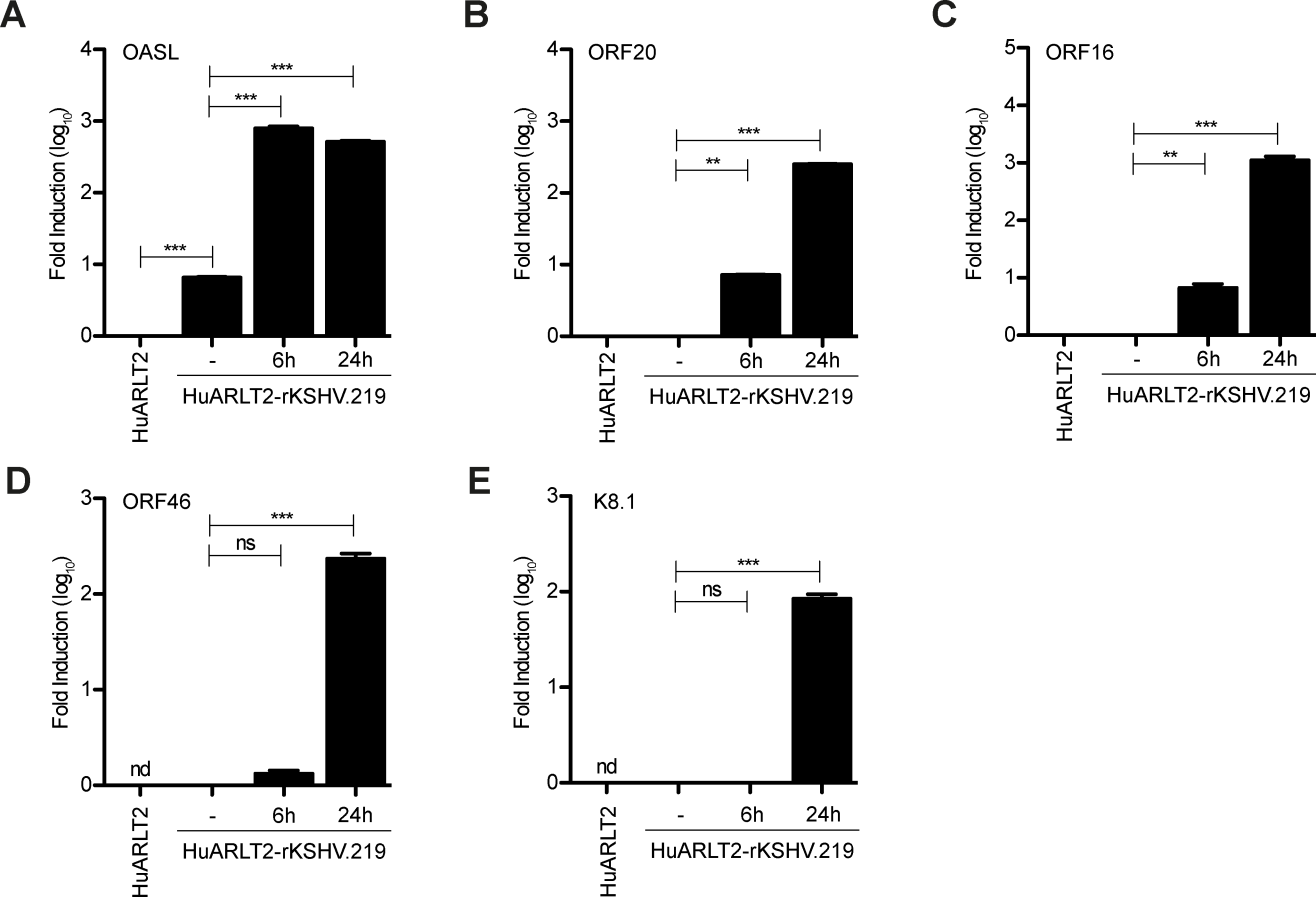
OASL and ORF20 are expressed in reactivated HuARLT2-rKSHV.219 cells. Uninfected HuARLT2 and latently infected HuARLT2-rKSHV.219 cells were seeded in 6-well plates. HuARLT2-rKSHV.219 cells were untreated (-) or reactivated by addition of sodium butyrate and RTA-expressing baculovirus, then incubated for the indicated times. RNA was prepared and (A) OASL (B) ORF20 (C) ORF16 (D) ORF46 or (E) K8.1 mRNA levels were determined by q-RT-PCR. The averages + SD of duplicates from 1 representative of 3 total experiments are shown. nd, not detected, ns, not significant, ** P<0.01, ***P<0.001.

OASL is expressed upon de novo infection with KSHV [38], but to date, the expression of OASL upon reactivation of KSHV-infected cells has not been well characterized. To determine whether OASL is expressed upon reactivation, we treated HuARLT-rKSHV.219 cells with sodium butyrate and RTA-expressing baculovirus to induce lytic reactivation, then collected samples for RNA isolation at 6 and 24 h. As a control, we included uninfected HuARLT2 cells. We then measured OASL and lytic transcript levels by q-RT-PCR. Compared to uninfected cells, OASL mRNA was increased in latently infected cells (Fig 11A). Upon reactivation, OASL mRNA expression significantly increased (Fig 11A). To analyze expression of lytic viral genes, we compared mRNA levels of ORF20, ORF16, OF46, and K8.1 in latently infected and reactivated cells. Based on *de novo* infection of endothelial cells and reactivation of latently infected BCBL-1 cells, ORF16 is classified as immediate early, ORF46 as early, K8.1 as late, and ORF20 as late [11, 39, 40]. We found that in HuARLT2-rKSHV.219 cells, ORF20 (Fig 11B) and ORF16 (Fig 11C) expression was already increased as early as 6h post reactivation, with expression further increasing at 24h. In contrast, very little ORF46 (Fig 11D) or K8.1 (Fig 11E) was detected at 6h post reactivation, but levels were greatly increased at 24h. Our data show that OASL and ORF20 mRNA expression are increased concomitantly during reactivation of HuARLT2-rKSHV.219 cells.

In summary, we have identified the cellular protein OASL as an interacting partner of KSHV ORF20 by q-AP-MS and identified novel interactions of ORF20 and OASL with ribosomal and nucleolar proteins. We characterized the conserved interaction of ORF20 isoforms and homologs with OASL, and we used a variety of mutants to further characterize the interaction of ORF20 with OASL. We found that both ORF20 and OASL co-sediment with ribosomal subunits. While ORF20 did not globally enhance translation, ORF20 expression strongly increased expression of endogenous OASL in an IRF3-dependent manner. Both OASL and ORF20 are expressed upon lytic reactivation of cells latently infected with KSHV. Finally, the presence of OASL enhances KSHV infectivity in an ORF20-dependent manner, suggesting a novel viral mechanism for usurping control of the host cell environment.

## Discussion

In this study, we have characterized the three protein isoforms of KSHV ORF20WT: ORF20FL, ORF20A, and ORF20B. We have found that like ORF20WT [10], all three ORF20 isoforms predominantly localize to the nuclei and nucleoli of transiently transfected cells. ORF20 is classified as a late lytic protein based on detection of ORF20 mRNA at late time points post *de novo* infection of endothelial cells or lytic reactivation of BCBL-1 cells [40] [11, 39]. We analyzed the expression of ORF20 mRNA upon lytic reactivation of endothelial cells, and found that it was expressed similarly to ORF16, which is classified as an immediate-early gene. This difference in expression may be due to reactivation versus *de novo* infection of endothelial cells, or due to differences in expression upon reactivation of endothelial versus B cells. This is not unusual for KSHV proteins; for example, K15 expression is very different in B cells and endothelial cells [29]. ORF20 is a member of the herpesviral UL24 family and the subcellular localization of all ORF20 isoforms was similar to the localization reported for multiple members of the UL24 family [9, 10].

As ORF20 is a poorly characterized protein, we wanted to better understand its function. We utilized an unbiased q-AP-MS approach to identify specific interacting partners of ORF20. During the course of our experiments, a global mapping study identified interacting partners of 89 KSHV proteins [41]. The authors identified high-confidence interactions using a mass spectrometry interaction statistics scoring algorithm that they developed, and identified one interacting partner for KSHV ORF20: coiled-coil domain containing protein 86 (CCDC86), also known as cytokine-induced protein with coiled-coil domain or cyclon [41]. The function of CCDC86 is not well understood. It localizes to the nuclei and nucleoli, is induced by IL-3 expression, and is involved in maintenance of T-cell homeostasis [42–44]. In our analysis of ORF20 interacting partners, we did not identify CCDC86. This may be due to differences in the cell type used, as we used HeLa S3, not 293T cells, or differences in the analysis method, as we used stable isotope labeling in cell culture for our q-AP-MS.

We found that ORF20 specifically interacted with the ISG oligoadenylate synthetase-like protein OASL. This is the first known interaction of a viral protein with OASL. We characterized the interaction of ORF20WT with a variety of OASL mutants and found that the interaction occurred independently of RNA-binding and the ubiquitin-like domain of OASL. Whether nucleolar localization of OASL is exclusively required for its interaction with ORF20WT is still unclear, as the expression of the OASLΔUBL mutant was low and thus nucleolar localization may have been obscured. We used C-terminal truncation mutants of the shortest ORF20 isoform, ORF20B, and found that the shortest mutant, ORF20B 1–186, was still able to bind wild-type OASL and localize to nucleoli.

Although the role of OASL during KSHV infection has not previously been studied directly, microarray analysis of cellular mRNA expression upon KSHV infection has shown that OASL expression is induced at late time points upon *de novo* KSHV infection [38]. We now show that OASL mRNA expression is increased in latently infected HuARLT2-rKSHV.219 cells compared to uninfected cells, and OASL mRNA expression is further increased upon reactivation with concomitant ORF20 mRNA expression. The exact mechanism of OASL induction by *de novo* KSHV infection or reactivation is not known, but OASL is induced by Sendai and influenza infection in an IRF3-dependent manner [13]. OASL is also expressed as an ISG downstream of the type I IFN receptor [13].

Until now, the effect of OASL on gammaherpesviral infection was not studied. When we infected OASL-reconstituted cells with MHV68-GFP or KSHV_LYT_, we found that the number of infected cells was significantly increased compared to EV-transfected cells. When we infected hOASL-reconstituted 293T OASL^-/-^ cells with KSHV_LYT_ ORF20stop, we did not observe enhancement of infection. However, when we infected OASL-reconstituted OASL^-/-^ cells with VSV-GFP, we observed inhibition of infection as expected. Taken together, these results indicate a unique proviral role for OASL during gammaherpesviral infection.

Two previous studies have analyzed protein-protein interactions of OASL and identified interactions with the transcriptional repressor methyl CpG binding protein 1 (MBD1) and RIG-I [24] [18]. However, no q-AP-MS data was available. When we analyzed the OASL interactome, we found that it was very similar to what we identified for ORF20, and in agreement with their nucleolar localization. We found that 40S and 60S ribosomal proteins were robustly represented among the identified interaction partners of both ORF20 and OASL. Ribosomal proteins are often considered contaminants in standard affinity purification mass spectrometry analysis and many ribosomal proteins are listed in the “contaminant repository for affinity purification”, or CRAPome [45]. However, q-AP-MS allows identification of ribosomal proteins as specific interaction partners. True interaction partners are identified by their specific abundance ratio for the bait protein, while non-specific contaminants can be identified based on their equal binding affinity for the bait and control proteins. The identification of ribosomal proteins for both ORF20 and OASL is thus of interest for elucidation of the functions of ORF20 and OASL.

To further characterize the interactions of ORF20 and OASL with ribosomes, we utilized a ribosomal sedimentation assay to analyze the association of both proteins with ribosomes. Both proteins sedimented with 40S and 60S ribosomal subunits when expressed individually and in combination with each other, further validating the protein interactions we identified by q-AP-MS. Interestingly, we found that when ORF20 and OASL were co-expressed, both proteins associated with polysomes. This may be due to increased expression when both were co-expressed, although recruitment to polysomes cannot be excluded. As association with polysomes may globally or selectively affect translation, we used a puromycin termination assay to examine the effect of ORF20 and OASL on cellular translation [34]. We found that puromycin was incorporated similarly in all samples. Taken together, the association with polysomes and similar incorporation of puromycin suggest selective control of gene expression. We found that endogenous OASL and ORF20 mRNA expression were increased concomitantly during reactivation of HuARLT2-rKSHV.219 cells. Additionally, we found that the OASL mRNA level was further upregulated upon RIG-I N overexpression or stimulation of endogenous RIG-I when ORF20 was present. We used siRNA knockdown to analyze the signaling components required for upregulation of OASL mRNA, and found that upregulation of OASL mRNA levels by ORF20 was IRF3- but not IFNAR-dependent. It is plausible that ORF20 enhances OASL expression at the mRNA level to allow sufficient protein levels for ORF20 to utilize OASL.

Regulation of gene expression occurs at many levels in eukaryotic cells. Transcriptional and post-transcriptional regulation are two major factors. Additionally, emerging evidence suggests that rather than possessing intrinsic translational capabilities, ribosomes contribute to control of gene expression. A recent publication has shown that translating ribosomes vary in their ribosomal protein composition, bestowing translational selectivity [46]. There are at least 80 different ribosomal proteins, and furthermore ribosomal RNA bases and ribosomal proteins can be modified. The number of possible ribosomes is therefore enormous [47]. Viral modulation of ribosome formation to form specialized ribosomes is one potential mechanism that viruses could use to enhance production of viral proteins and necessary cellular factors. By manipulating the incorporation of specific ribosomal proteins, a virus could promote translation of desirable mRNAs. It is possible that ORF20 increases expression of OASL to allow both proteins to contribute to formation of specialized ribosomes and translational control, which is an exciting topic for future study.

## Materials and methods

### Cell culture

Human embryonic kidney 293T (CRL-3216), human embryonic kidney 293 (CRL-1573), primary human foreskin fibroblast HFF-1 (SCRC-1041), human epithelial adenocarcinoma HeLa (CCL-2) and HeLa S3 (CCL-2.2), immortalized retinal pigment epithelial hTERT RPE-1 (CRL-4000), murine bone marrow fibroblast M2-10B4 (CRL-1972), African green monkey kidney Vero (CCL-81), and baby hamster kidney BHK-21 (CCL-10) cells were originally obtained from the American Type Culture Collection (ATCC). 293T OASL^-/-^ and corresponding control 293T cells were kindly provided by Veit Hornung (Ludwig Maximilians Universität, Munich, Germany) [18]. Conditionally immortalized human endothelial cells stably infected with rKSHV.219, HuARLT2-rKSHV.219, and corresponding uninfected HuARLT2 control cells [29, 31, 48] were kindly provided by Thomas F. Schulz (Hannover Medical School, Hannover, Germany). HuARLT-rKSHV.219 and HuARLT2 cells were maintained in Cellovations enhanced microvascular endothelial cell growth medium (PB-MH-100-4099, PELOBiotech, Planegg, Germany) supplemented with 2 μg/ml doxycycline and for HuARLT2-rKSHV.219 additionally 5 μg/ml puromycin. All other cell lines were maintained in basal medium of Gibco high glucose (4.5 g/L) DMEM containing L-glutamine (ThermoFisher Scientific) supplemented with 8% fetal calf serum (FCS), penicillin/streptomycin (p/s), and sodium pyruvate; medium for HeLa and HeLa S3 additionally included 1% non-essential amino acids (NEAA), and medium for M2-10B4 and BHK-21 cells lacked sodium pyruvate. hTERT-RPE1 were cultured in high glucose DMEM with 5% FCS and p/s. HEK 293 were cultured in high glucose DMEM with 10% FCS and p/s. HFF-1 were cultured in high glucose DMEM supplemented with 15% FCS and p/s. Vero cells were cultured in MEM supplemented with 7.5% FCS, glutamine, NEAA, and p/s. All cell lines were cultured at 37°C in a humidified, 7.5% CO_2_ environment.

### Antibodies

Antibodies against fibrillarin (# 2639), IRF3 (# 4302 for immunoblotting), STAT1 (# 9172), and the anti-myc tag mouse monoclonal 9B11 (# 2276) were from Cell Signaling. Rabbit anti-c-myc (# C3956), mouse anti-tubulin (# T6199-200UL), and mouse anti-beta-actin (#A5441) antibodies were purchased from Sigma-Aldrich. The anti-puromycin antibody clone 12D10 (# MABE343) was purchased from Merck Millipore. Anti-V5 antibodies were purchased from Invitrogen (# R960-25) and Biolegend (# 680601). Rabbit anti-GFP antibody ab290 was purchased from Abcam and HRP-coupled mouse anti-GFP antibody (sc-9996 HRP) and anti-IRF3 (sc-9082 for immunofluorescence) were purchased from Santa Cruz. Two rabbit polyclonal antibodies, anti-OASL 7 and anti-OASL 8, were raised in individual rabbits against a C-terminal peptide of OASL, KQQIEDQQGLPKKQ, which corresponds to amino acids 460–473 within the OASL ubiquitin-like domain.

### Plasmids

pcDNA4/myc-His B, pcDNA4/myc-His/LacZ (LacZ-myc), and pcDNA3.1(+) were purchased from Invitrogen. The LacZ-myc plasmid encodes the beta-galactosidase protein and is referred to by the LacZ construct name throughout the text. ORF20WT was cloned into pcDNA4/myc-His B and encodes genomic KSHV ORF20 with a C-terminal myc-tag [10]. The ORF20WT plasmid construct can potentially express myc-tagged ORF20FL from its ATG (methionine) start codon at nucleotide position (nt) 1, myc-tagged genomic ORF20A (gA) from its CTG (leucine) start codon at nt 70, and myc-tagged ORF20B from its ATG start codon at nt 190. pcDNA4/myc-His B constructs to express myc-tagged ORF20FL, ORF20A, or ORF20B individually were made using ORF20WT as a template and utilized site-directed mutagenesis and/or PCR based cloning. To enhance expression of ORF20A individually, an ATG was added 5’ of the genomic leucine CTG start codon. pcDNA4/myc-His B constructs expressing two myc-tagged isoforms were also made using mutagenesis and/or PCR based cloning. ORF20FLgA and ORF20FLB potentially express ORF20FL and ORF20A or ORF20FL and ORF20B, respectively, from their genomic start codons. ORF20AB, which can express ORF20A and ORF20B, includes the genomic ORF20B start codon and a 5’ ATG upstream of the genomic ORF20A start to enhance plasmid-based expression.

MHV68 ORF20 (WUMS strain, NCBI NC_001826.2) with two sequential C-terminal myc tags was codon optimized for human expression and synthesized by Integrated DNA Technologies, then cloned into pcDNA4/myc-His B to make an MHV68 ORF20 construct with three C-terminal myc tags. Other homologs were cloned into pcDNA4/myc-His B and have one C-terminal myc tag. MCMV M76-myc (MCMV Smith strain, NCBI GU305914.1) and HSV-1 UL24-myc (NCBI NC_001806.1) were cloned using plasmid templates and HCMV UL76-myc (NCBI AY446894.2) was cloned by direct PCR of an *E*. *coli* colony containing the HCMV Merlin BAC, kindly provided by Martin Messerle (Hannover Medical School, Germany) [49]. pEGFP-RIG-I, expressing a GFP-RIG-I fusion, and pCAGGS Flag-RIG-I N (RIG-I N), expressing a constitutively active truncation mutant of RIG-I, were kindly provided by Andreas Pichlmair (Technical University of Munich, Germany). pCMV GFP-NS1A(203–237), referred to as GFP-NS1A, expresses GFP fused to amino acids 203–237 of influenza A/Udorn/72 encoding a nucleolar localization sequence and localizes to the nucleoli of transfected cells [33]. GFP-NS1A was kindly provided by Ilkka Julkunen (University of Turku, Finland). pcDNA3.1 human OASL-V5 WT (OASL-V5 or hOASL) and mutants have been previously described [17, 21]. pcDNA3.1 murine OASL1-V5 (mOASL1) encodes the murine homolog of human OASL (mOASL1: NM_145209.3). pcDNA3.1(+) OASL-myc (OASL-myc) expresses human OASL with a C-terminal myc tag. When not otherwise specified in the text, OASL refers to hOASL. ORF20WT-myc, ORF20B-myc, ORF20FL-myc, and OASL-V5 were subcloned into the lentiviral expression vector pWPI puro, kindly provided by Didier Trono, and lentiviral particles were prepared using pCMV gag-pol and pVSV-G.

Inserts of novel constructs were fully sequenced; other constructs were partially sequenced for verification. Primer and cDNA sequences are available upon request.

### BAC mutagenesis for construction of ORF20stop

We used *en passant* BAC mutagenesis [50] to construct an ORF20stop mutant on the constitutively lytic KSHV (KSHV_LYT_) backbone [36, 37]. We mutated the ORF20FL GAG codon for E69 into the stop codon TAG to stop translation of ORF20FL, ORF20A, and ORF20B without altering the ORF21 promoter. KSHV_LYT_-BAC mutant clones were fully sequenced to confirm successful mutagenesis and to exclude additional undesired mutations in the viral genome.

### Virus stock preparation

BAC-derived MHV68-GFP [51] was amplified in M2-10B4 cells, concentrated by centrifugation for 2 h at 26,000 ×*g* and 4°C, purified by pelleting through a 10% Nycodenz cushion for 3 h at 36,000 ×*g*, and resuspended in sterile virus standard buffer (50 mM Tris-HCl, pH 7.8, 12 mM KCl, 5 mM Na_2_-EDTA) essentially as previously described [36]. For reconstitution of KSHV_LYT_ wildtype or ORF20stop, BAC DNA was transfected into hTERT RPE-1 cells using Polyfect (Qiagen), propagated in the same cells, and concentrated by centrifugation for 4 h at 15,000 ×*g* at 4°C. Viral stock titers were determined using the median tissue culture infective dose (TCID_50_) method on M2-10B4 cells and hTERT-RPE1 cells for MHV68 and KSHV_LYT_, respectively, as previously described [36, 37]. Vesicular stomatitis virus with GFP inserted between the G and L genes [52] was kindly provided by Andrea Kröger (Otto von Guericke University Magdeburg, Germany). VSV-GFP was amplified in BHK-21 cells and the stock titer was determined by plaque assay on Vero cells. Lentiviral particles were prepared by co-transfection of low-passage 293T cells in 6 well plates with 2 μg of the appropriate pWPI expression vector, 1.3 μg pCMV gag-pol, and 700 ng VSV-G; DNA was complexed with 10 μl Lipofectamine 2000.

Approximately 16 h post transfection, medium was changed to viral harvest medium (high glucose DMEM containing 20% FCS, p/s, and 10 mM HEPES). The following day, approximately 40–44 h post transfection, supernatants were filtered through 0.45 μm filters and used immediately for transduction.

### Immunoprecipitations

Transfected cells were lysed in mild NP-40 lysis buffer, containing 50 mM Tris-HCl, pH 7.4, 150 mM NaCl, 1% IGEPAL CA-630 (NP-40 substitute), 0.25% sodium deoxycholate, and 1x complete protease inhibitors without EDTA (Roche), or stringent radioimmunoprecipitation (RIPA) buffer containing 20 mM Tris-HCl, pH 7.5, 100 mM NaCl, 1 mM EDTA, 1% Triton X-100, 0.5% sodium deoxycholate, 0.1% SDS, and 1x complete protease inhibitors (Roche). One-tenth of the lysate was reserved as input lysate, the remainder was pre-cleared with protein A agarose beads (Repligen), immunoprecipitated (IP) with anti-myc, anti-V5, or anti-GFP antibodies, and then incubated with protein A agarose beads. For anti-FLAG IPs, anti-FLAG antibody and Pierce protein G magnetic beads (ThermoFisher) were used. Following extensive washing, bound protein was eluted by heating samples in Laemmli or NuPAGE sample buffer. Input lysates and IP samples were then processed for immunoblotting.

### Immunoblotting

Cell lysates were combined with Laemmli or NuPAGE sample buffer, heated, and separated by SDS-PAGE or Bolt Bis-Tris Plus PAGE (Thermo Fisher Scientific). Both methods were used interchangeably for most proteins; for endogenous OASL, exclusively Bis-Tris PAGE was used. Proteins were transferred to nitrocellulose or PVDF membranes using wet transfer and either Towbin or NuPAGE transfer buffer, as appropriate. After blocking with 5% milk in TBST, membranes were incubated with appropriate primary and secondary antibodies, developed using Lumi-Light (Roche Applied Science) or SuperSignal West Femto (Thermo Scientific) chemoluminescence substrates, and signal was detected using either film exposure or an Intas Chemidoc system.

### Transduction

HFF, HuARLT2-rKSHV.219, or HuARLT2 were seeded in 6 well plates, then transduced with 1 ml filtered lentiviral-containing supernatant, 2 ml appropriate cell-specific medium, and 8 μg/ml polybrene. Plates were centrifuged at 690 × *g* for 90 min at 30°C, incubated 1–3 h at 37°C, then medium was exchanged. After 4 days, cells were seeded onto glass coverslips.

### Immunofluorescence microscopy

HeLa cells were transfected in plastic multiwell plates and seeded onto glass coverslips approximately 24 h post transfection. For whole-cell staining, cells were fixed with 4% paraformaldehyde in PBS (PFA) approximately 48 h post transfection. For nuclear staining, 48 h post transfection, HeLa cells were washed once with PBS, incubated 5 minutes in cold 1% NP-40 extraction buffer (50 mM Tris-HCl, pH 7.5, 150 mM NaCl, 1% NP-40), washed once with TBS, then fixed with 3% PFA [53]. HeLa cells and nuclei on coverslips were washed, blocked and permeabilized with 5% FCS and 0.3% Triton-X 100 in PBS, then stained with the indicated primary antibodies, followed by staining with Alexa Fluor 488 or Alexa Fluor 594 conjugated secondary antibodies. Antibodies were diluted in PBS containing 0.1% BSA and 0.3% Triton-X 100. Transiently transduced HFF, HuARLT2-rKSHV.219, and HuARLT2 cells were seeded onto glass coverslips. For reactivation, cells were treated with 2μM sodium butyrate and 10% SF-9 supernatant containing RTA-expressing baculovirus [29, 32]. Cells were fixed with 4% PFA at various times post seeding, then processed essentially as for HeLa cells, with the addition of Alexa Fluor 647 conjugated secondary antibodies. 293T or 293T OASL^-/-^ cells were seeded onto glass coverslips, then transfected with ORF20WT-myc with or without RIG-I N. Cells were fixed with 4% PFA, permeabilized with 0.1% Triton X-100, blocked in PBS containing 10% FCS and 1% BSA, then stained with anti-myc and/or anti-IRF3 antibodies. HeLa S3 cells were seeded onto glass coverslips. The following day, they were transfected with ORF20WT-myc or OASL-V5 individually, or co-transfected with ORF20WT-myc and OASL-V5. 24 h post transfection, HeLa S3 cells were fixed with methanol and PFA, blocked in PBS containing 10% FCS and 1% BSA, then stained with primary and secondary antibodies diluted in PBS containing 1% BSA. All nuclei were counterstained with Hoechst. Images were obtained using a Nikon ECLIPSE Ti-E inverted microscope equipped with a spinning disk device (Perkin Elmer Ultraview) and images were processed using Volocity (Improvision) and Adobe Photoshop.

### Quantitative affinity purification coupled to mass spectrometry (q-AP-MS)

q-AP-MS to analyze protein-protein interactions was performed [54]. HeLa S3 cells were metabolically labeled for 10 days in lysine- and arginine-free DMEM (Pierce) supplemented with dialyzed fetal bovine serum (Pierce), p/s (Invitrogen), 2 mM glutamine (Invitrogen), and either 0.22 mM ^13^C_6_^15^N_2_ L-lysine-2HCl and 0.1385 mM ^13^C_6_ L-arginine-HCl (Pierce) for heavy-labeled cells or the corresponding unlabeled amino acids (Pierce) for light-labeled cells. At day 10, approximately 7x10^6^ heavy- or light-labeled HeLa S3 cells were plated into two 14 cm dishes each. The following day, heavy and light HeLa S3 cells were transfected with 48.5 μg DNA and 100 μl Lipofectamine 2000 (Life Technologies) diluted in arginine- and lysine-free medium. For the forward experiment, heavy labeled HeLa S3 cells were transfected with ORF20WT or OASL-myc, while light labeled HeLa S3 cells were transfected with LacZ-myc. In the crossover experiment, the cell labels were inverted.

24–36 h post transfection, cells were carefully washed with ice-cold PBS and then lysed in 1 ml NP-40 lysis buffer (see recipe under **Immunoprecipitations**) containing 50U Emprove bio benzonase (Merck #1.01695.0001) for 1 hour at 4°C. Clarified supernatant was immunoprecipitated with 75 μl anti-c-myc microbeads (Miltenyi Biotec) for 1 h at 4°C on a rotating platform. Forward samples were combined or crossover samples were combined and then immediately applied to equilibrated M columns in a μMACS separator (Miltenyi Biotec). Beads were washed four times with 200 μl NP-40 lysis buffer, then twice with 200 μl Miltenyi wash buffer 2. Bound proteins were eluted by 3 × 10 min incubations with 100 μl of 100 mM glycine, pH 2.5; all fractions were combined. Eluted protein was precipitated by sequential addition of 70 μl 2.5M sodium acetate, pH 5.0, 40 μl 20 mM Tris-HCl, pH 8.2, 1 μl Glycoblue (Ambion), and 1600 μl 100% ethanol, followed by overnight incubation at 4°C on a rocking platform. Proteins were pelleted by centrifugation at 17,949 ×*g* for 60 min at 4°C, the supernatant was removed, and the protein pellet was air dried at room temperature.

Later, the protein pellet was rehydrated, disulfide bonds were reduced and cysteines blocked and alkylated, and an in-solution digest was performed using Lys-C and trypsin. Peptides were further processed and analyzed by liquid chromatography coupled to tandem mass spectrometry (LC-MS/MS) using a Dionex UltiMate 3000 n-RSLC low flow liquid LC system (Thermo Scientific) connected to an LTQ Orbitrap VelosPro mass spectrometer (Thermo Scientific).

Peptides were identified using Proteome Discoverer 1.3.0.339 and a human protein database extracted from SwissProt on a Mascot server (V 2.4, Matrix Science) with ORF20 and LacZ manually added. The following search parameters were used: enzyme, trypsin; maximum missed cleavages, 1; fixed modification, methyl methane thiosulfonate (C); variable modification, oxidation (M); peptide tolerance, 5 ppm; MS/MS tolerance, 0.5 Da, Arg6 and Lys8 as variable modifications for quantification. Peptide filter parameters were as follows: maximum peptide rank of 1, peptide confidence of medium, and Mascot ion score of at least 25. The log_2_ fold change values of heavy/light values of each identified protein were calculated to facilitate graphical visualization. Highly confident interaction partners were based on log_2_ fold change values with an absolute value ≥1 in one experiment and ≥ 0.7 in the other experiment. Highly confident interaction partners for ORF20-myc and OASL-myc were entered into VennDis to identify specific and shared proteins and to create a Venn diagram.

### Sucrose gradient fractionation of ribosomes

293T cells were transfected with either ORF20WT, OASL-myc, or both combined. 24 h post transfection, sucrose gradient fractionation of ribosomes was performed essentially as described [55, 56]. Cell lysates (50 mM Tris-HCl, pH 7.6, 150 mM NaCl, 1 mM DTT, 1% NP-40 substitute, 100 μg/ml cycloheximide, DNase I, RNAse inhibitor, and protease inhibitor) were prepared in the presence of 20 mM EDTA to disrupt ribosomes and polysomes or in the presence of 10 mM MgCl_2_ to stabilize ribosomes and polysomes. Lysates were centrifuged over a 5–45% linear sucrose gradient at 160,000 ×*g* in a Beckman SW40Ti rotor for 3 h at 4°C. Fractions were collected from the top using a Biocomp fractionator (Biocomp, NB Canada) and absorbance at 254 nm was measured. RNA and total protein were extracted from 350 μl of each fraction by using Trizol reagent (ThermoFisher Scientific) and subjected to denaturing agarose gel electrophoresis or 10% SDS-PAGE and immunoblotting as appropriate.

### Puromycin incorporation assay

293T cells were transfected with pcDNA4/myc-His B (EV), ORF20WT, ORF20FL, or ORF20B, and a small amount of either pcDNA3.1(+) (EV, control) or RIG-I N to induce expression of endogenous OASL. 24 h post transfection, newly synthesized proteins were labeled with puromycin by treating cells for 15 minutes with 5 μg/ml puromycin [34]. Cells were immediately lysed in 200 μl 1× NuPAGE LDS sample buffer supplemented with 3% beta-mercaptoethanol, 250 U benzonase, and 5 mM MgCl_2_. Lysates were incubated for 30 minutes at room temperature, denatured for 10 minutes at 70°C, separated on 4–12% Bolt Bis-Tris plus gradient gels (ThermoFisher Scientific) with NuPAGE MOPS SDS running buffer (50 mM MOPS, 50 mM Tris base, 0.1% SDS, 1 mM EDTA), and transferred to 0.2 μm PVDF membranes. Anti-puromycin, anti-OASL, anti-myc, and anti-actin immunoblotting were performed sequentially. Between antibody incubations, membranes were treated with Restore western blot stripping buffer (ThermoFisher) according to the manufacturer’s instructions.

### Quantitative reverse transcriptase polymerase chain reaction

293 cells in 12-well plates or 293T cells in 6-well plates were transfected with the indicated plasmids for 24–48 h. For 293 cells, 24 h post transfection, cells were treated with Lipofectamine or transfected with 5’pppRNA complexed with Lipofectamine for 24 h. RNA was prepared using commercially available kits. cDNA was synthesized using anchored-oligo(dT)_18_ and the Transcriptor first strand cDNA synthesis kit (Roche) or the iScript cDNA synthesis kit (Biorad). The quantity of GAPDH, OASL, IRF3, IFNAR, STAT1, ORF20, ORF16, ORF46, or K8.1 cDNA was determined using the LightCycler 480 Sybr Green I Master 2× mix (Roche), 125 nM of gene-specific forward and reverse oligonucleotides, and 1 μl cDNA, or the GoTaq 2x qPCR master mix, 200 nM of gene-specific forward and reverse oligonucleotides, and 1 μl cDNA, in a Roche LightCycler 480 instrument. mRNA levels were quantified relative to GAPDH and the 2^-ΔΔ*C*T^ method [57] was used to compare the amount of the indicated mRNA between samples. The forward and reverse oligonucleotide sequences were GAAGGTGAAGGTCGGAGTC and GAAGATGGTGATGGGATTTC for GAPDH, GCCATGTACTCCAGAACTCATC and GGCCTGGGATAACTCATTGTAA for OASL, AGCCTCGAGTTTGAGAGCTA and TGGTCCGGCCTACGATG for IRF3, CACCATTTCGCAAAGCTCAG and ACCATCCAAAGCCCACATAA for IFNAR, GCTGCAGAACTGGTTCACTAT and GGGTCATGTTCGTAGGTGTATTT for STAT1, CGATCTATGGCGGTTTCTAAGT and TTACGCAGTCGGCAATTCT for ORF20, AGATTTCACAGCACCACCGGTA and CCCCAGTTCATGTTTCCATCGC for ORF16, CACTGCTGCGATCCAGAGGATA and GAACCTGACATTGCGGATCCAC for ORF46, and TAAACGGGACCAGACTAGCAGC and GTTTTCTGCGACCGGTGATACG for K8.1. The oligonucleotide sequences for ORF16, ORF46, and K8.1 were previously published [58].

### siRNA knockdown of IRF3, STAT1, or IFNAR1

All siRNAs were purchased from Dharmacon/GE Life Sciences: ON-TARGETplus non-targeting pool (D-001810-10-20, control siRNA), ON-TARGETplus human IRF3 SMARTpool siRNA (L-006875-00-0010), ON-TARGETplus human STAT1 SMARTpool siRNA (L-003543-00-0005), and ON-TARGETplus human IFNAR1 SMARTpool siRNA (L-020209-00-0005). 293T cells were reverse transfected with siRNAs complexed with Lipofectamine 2000. Per well of a 6 well plate, 1 μl of 50μM siRNA stock was combined with 199 μl OptiMEM. Separately, 6 μl Lipofectamine 2000 was combined with 194 μl OptiMEM. Diluted Lipofectamine was added to diluted siRNA, then all 400 μl were added directly to the plate. 293T cells (450,000–600,000 cells) were resuspended in 1.6 ml/well, then added to the siRNA-Lipofectamine complexes. 2 days later cells were co-transfected with pcDNA4/myc-His B (EV) or ORF20WT, and either pcDNA3.1(+) (EV) or RIG-I N. 24 h later, RNA was prepared and q-RT-PCR was performed for OASL, GAPDH, and either IRF3, STAT1, or IFNAR as appropriate. In parallel, 293T cells were transfected with siRNAs for preparation of protein lysates and immunoblotting analysis.

### OASL reconstitution and infection assays

150,000 293T OASL^-/-^ cells were seeded per well in 24-well plates. The following day, replicate wells were transfected with either pcDNA3.1(+) (EV), mOASL1, or hOASL: 40μl of DNA mixes containing 748 ng DNA and 2.62μl Fugene HD in OptiMEM were added per well. Approximately 24 hours after transfection, one master dilution of the appropriate virus was prepared, then used to infect transfected cells with VSV-GFP for 1 hour, MHV68-GFP for 2 hours, or with KSHV_LYT_ wildtype or KSHV_LYT_ ORF20stop for 4 hours, at 37°C. Virus inoculum was removed, cells were washed once with medium, and fresh medium was added. The next day, 16 h, 20 h, or 24 h post infection for VSV-GFP, MHV68-GFP, or KSHV_LYT_, respectively, cells were detached using trypsin, fixed for 30 minutes with 4% PFA, and the number of green cells was determined by flow cytometry on an LSRII instrument. For growth curves with MHV68-GFP, at the indicated time points 10% of medium was harvested, frozen at -70°C, and replaced with fresh medium. After all samples were collected, the titers in supernatants were determined by TCID_50_ assay on M2-10B4 cells.

### Reactivation of HuARLT2-rKSHV.219 cells

1 day after seeding in Cellovations endothelial cell medium lacking selection antibiotics, HuARLT2-rKSHV.219 cells were reactivated by the addition of 2 μM sodium butyrate and 10% SF-9 supernatant containing KSHV RTA-expressing baculovirus, kindly provided by Thomas F. Schulz [29, 32]. Cells were incubated for various times, then fixed for immunofluorescence or lysed for preparation of RNA.

### Statistical analysis

Statistical analysis was performed using Graphpad Prism. Statistical significance was determined by 1way ANOVA followed by Tukey’s multiple comparison test (Fig 9B–9D, Fig 11, S6 Fig), 1way ANOVA followed by Dunnett’s multiple comparison test (Fig 10E), or by two-tailed unpaired t test (Fig 10B–10D, 10F and 10H). For all figures, ns indicates not significant, * indicates P<0.05, ** indicates P<0.01, and *** indicates P<0.001.

## Supporting information

S1 Fig Subcellular localization of ORF20 and OASL is cell-type independent. (A+B) HFF-1 (A) or HuARLT2 (B) were transiently transduced with lentiviruses encoding the indicated construct. Transduced cells were seeded onto coverslips, then processed for anti-myc (pink) immunofluorescence. (C) 293T or 293T OASL^-/-^ were transiently transfected with ORF20WT-myc, then processed for anti-myc (pink) immunofluorescence. (D) 293T cells were transiently transfected with EV or ORF20WT-myc and RIG-I N, then processed for anti-myc (pink) and anti-endogenous IRF3 (green) immunofluorescence. (A-D) Nuclei were counterstained with Hoechst. Scale bar = 10 μm
(TIF)

S2 Fig OASL-V5 co-immunoprecipitates with ORF20-myc-GFP but not nucleolar GFP-NS1A. (A, B) 293T cells were transfected with EV, two separate clones of ORF20-myc-GFP, RIG-I GFP, GFP-NS1A, and OASL-V5 or EV as indicated. (A) An anti-V5 immunoprecipitation of RIPA lysates was performed and input lysates and immunoprecipitates were immunoblotted with anti-GFP and anti-V5 antibodies. (B) An anti-GFP immunoprecipitation of RIPA lysates was performed and input lysates and immunoprecipitates were immunoblotted with anti-V5 and anti-GFP antibodies as indicated.
(TIF)

S3 Fig Amino acid sequence alignment of selected UL24 family members. The amino acid sequences of HSV-1 UL24, HCMV UL76, MCMV M76, KSHV ORF20WT (FL with genomic ORF20A and ORF20B start codons), KSHV ORF20A, KSHV ORF20B, and MHV68 ORF20 were aligned using Clustal W2.
(TIF)

S4 Fig OASL and most mutants localize to the cytoplasm and nucleoli of transfected cells. HeLa cells were transfected with the indicated plasmid and processed for whole cell and nuclear anti-V5 (green) and anti-fibrillarin (red) immunofluorescence. Nuclei were counterstained with Hoechst (blue). Images are representative of three independent experiments. Scale bar = 20 μm.
(TIF)

S5 Fig ORF20B mutants localize to the nuclei and nucleoli of transfected cells. HeLa cells were transfected with plasmids expressing the indicated myc-tagged ORF20B deletion mutant plasmid and processed for whole cell and nuclear anti-myc (green) and anti-fibrillarin (red) immunofluorescence. Nuclei were counterstained with Hoechst (blue). Images are representative of three independent experiments. Scale bar = 30 μm (whole cell IF) and 15 μm (nuclear IF)
(TIF)

S6 Fig Additional nuclear KSHV ORFs do not upregulate OASL induction and verification of siRNA knockdown. (A) 293T cells were co-transfected with the indicated plasmids for 24 h. The amount of OASL mRNA was determined by q-RT-PCR. (B, C, D) IRF3, IFNAR, or STAT1 mRNA levels were measured in the same samples described in Fig 9D. (A-D) Data shown are means + SD of duplicates from at least two experiments. Statistical significance was measured by one-way ANOVA followed by Tukey’s posttest ** P<0.01, *** P<0.001 (B, D) In parallel with preparation of samples for qPCR, protein lysates were prepared and analyzed for (B) IRF3 or (D) STAT1 expression by immunoblotting.
(TIF)

S7 Fig ORF20 does not affect the interaction between OASL and RIG-I or their co-localization. **(A)** 293T cells were transfected with the indicted combinations of FLAG-RIG-I, OASL-V5, ORF20WT-myc, and/or EV. NP40 lysates were subjected to anti-FLAG IP. Input lysates and immunoprecipitates were subjected to anti-FLAG, anti-V5, and anti-myc immunoblotting. (B and C) HeLa S3 cells on glass coverslips were transfected with the indicated plasmids, then processed for anti-FLAG, -V5, or -myc immunofluorescence as appropriate. Nuclei were counterstained with Hoechst. Scale bar = 20 μm.
(TIF)

S1 Dataset ORF20 interactome. Interacting partners of ORF20 were identified by q-AP-MS and data were analyzed using Proteome Discoverer. The data as exported from Proteome Discoverer, as well as annotated results, are provided.
(XLSX)

S2 Dataset OASL interactome. Interacting partners of OASL were identified by q-AP-MS and data were analyzed using Proteome Discoverer. The data as exported from Proteome Discoverer, as well as annotated results, are provided.
(XLSX)

S1 Supporting Information Highly confident interaction partners for ORF20 and OASL identified by q-AP-MS and comparison of specific and shared partners. This file shows the highly confident interaction partners for ORF20 and OASL identified by q-AP-MS (tabs: ORF20-myc partners and OASL-myc partners), taking into account the log_2_ fold change values and the H/L counts. A protein was characterized as highly confident if the log_2_ fold change had an absolute value ≥1 in one experiment and ≥0.7 in the other experiment. The transfected proteins (ORF20, OASL, and LacZ) were omitted, as were less confident interacting partners. The highly confident interaction partners were entered into VennDis to create a Venn Diagram. The proteins identified by VennDis as ORF20-specific, shared, and OASL-specific are listed (tab: specific and shared).
(XLSX)
