# Supplementary figures and images for: The interferon-stimulated gene product oligoadenylate synthetase-like protein enhances replication of Kaposi’s sarcoma-associated herpesvirus (KSHV) and interacts with the KSHV ORF20 protein
Source: PLoS Pathog. 2018 Mar 2;14(3):e1006937. doi: 10.1371/journal.ppat.1006937 (PMC5851652; doi:10.1371/journal.ppat.1006937)

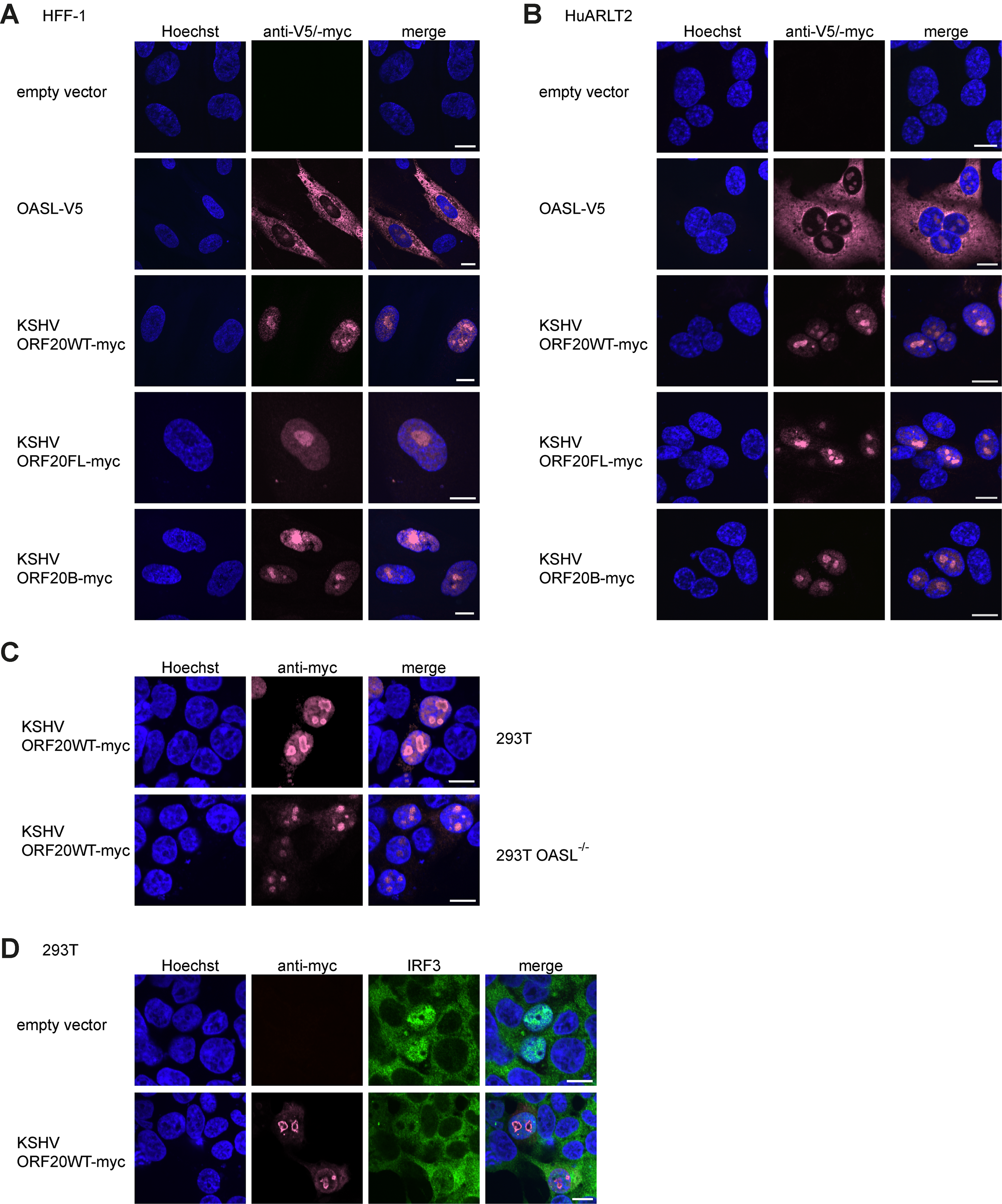

Supplement: S1 Fig — (A+B) HFF-1 (A) or HuARLT2 (B) were transiently transduced with lentiviruses encoding the indicated construct. Transduced cells were seeded onto coverslips, then processed for anti-myc (pink) immunofluorescence. (C) 293T or 293T OASL-/- were transiently transfected with ORF20WT-myc, then processed for anti-myc (pink) immunofluorescence. (D) 293T cells were transiently transfected with EV or ORF20WT-myc and RIG-I N, then processed for anti-myc (pink) and anti-endogenous IRF3 (green) immunofluorescence. (A-D) Nuclei were counterstained with Hoechst. Scale bar = 10 μm (TIF) [file ppat.1006937.s001.tif]

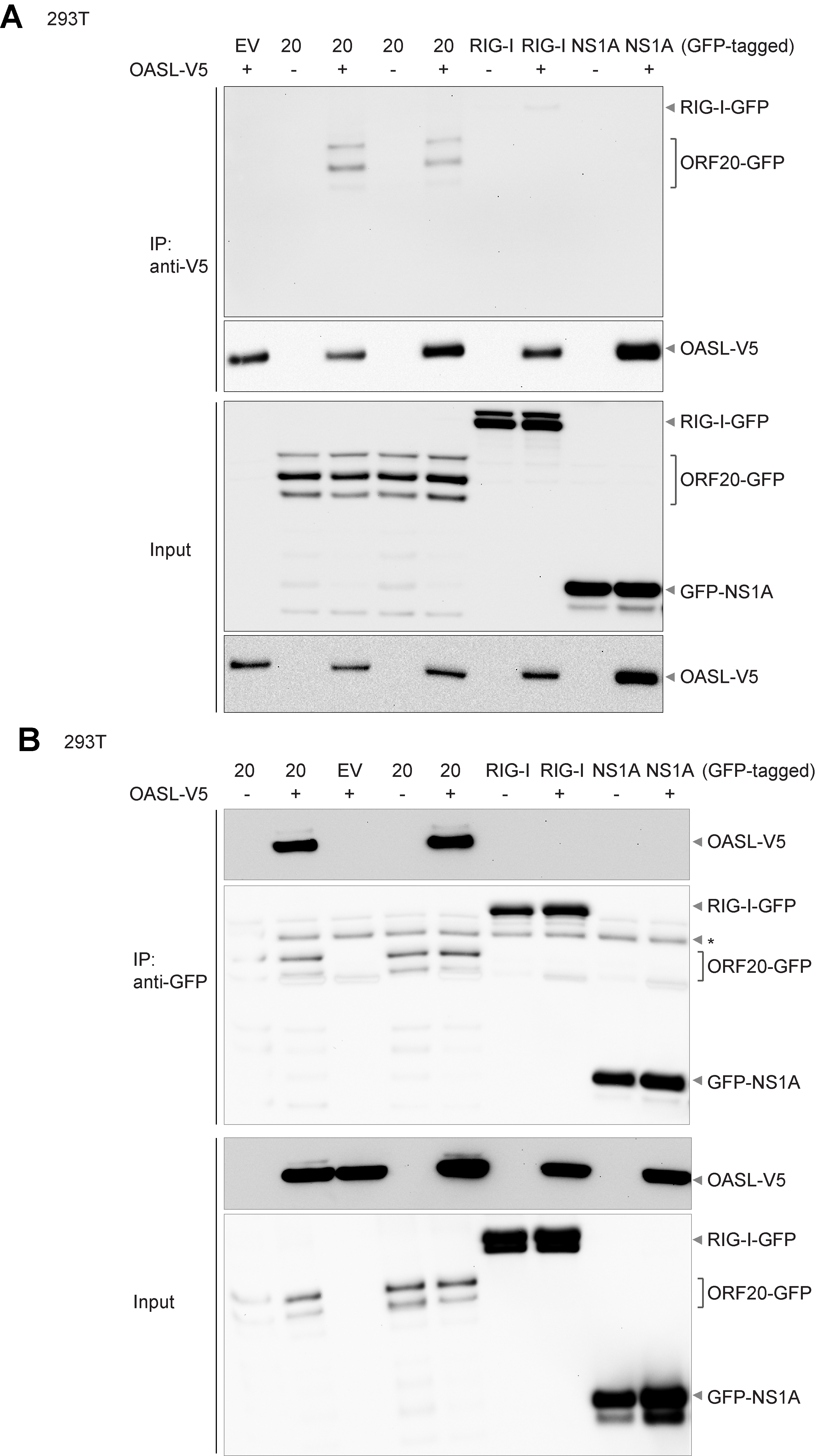

Supplement: S2 Fig — (A, B) 293T cells were transfected with EV, two separate clones of ORF20-myc-GFP, RIG-I GFP, GFP-NS1A, and OASL-V5 or EV as indicated. (A) An anti-V5 immunoprecipitation of RIPA lysates was performed and input lysates and immunoprecipitates were immunoblotted with anti-GFP and anti-V5 antibodies. (B) An anti-GFP immunoprecipitation of RIPA lysates was performed and input lysates and immunoprecipitates were immunoblotted with anti-V5 and anti-GFP antibodies as indicated. (TIF) [file ppat.1006937.s002.tif]

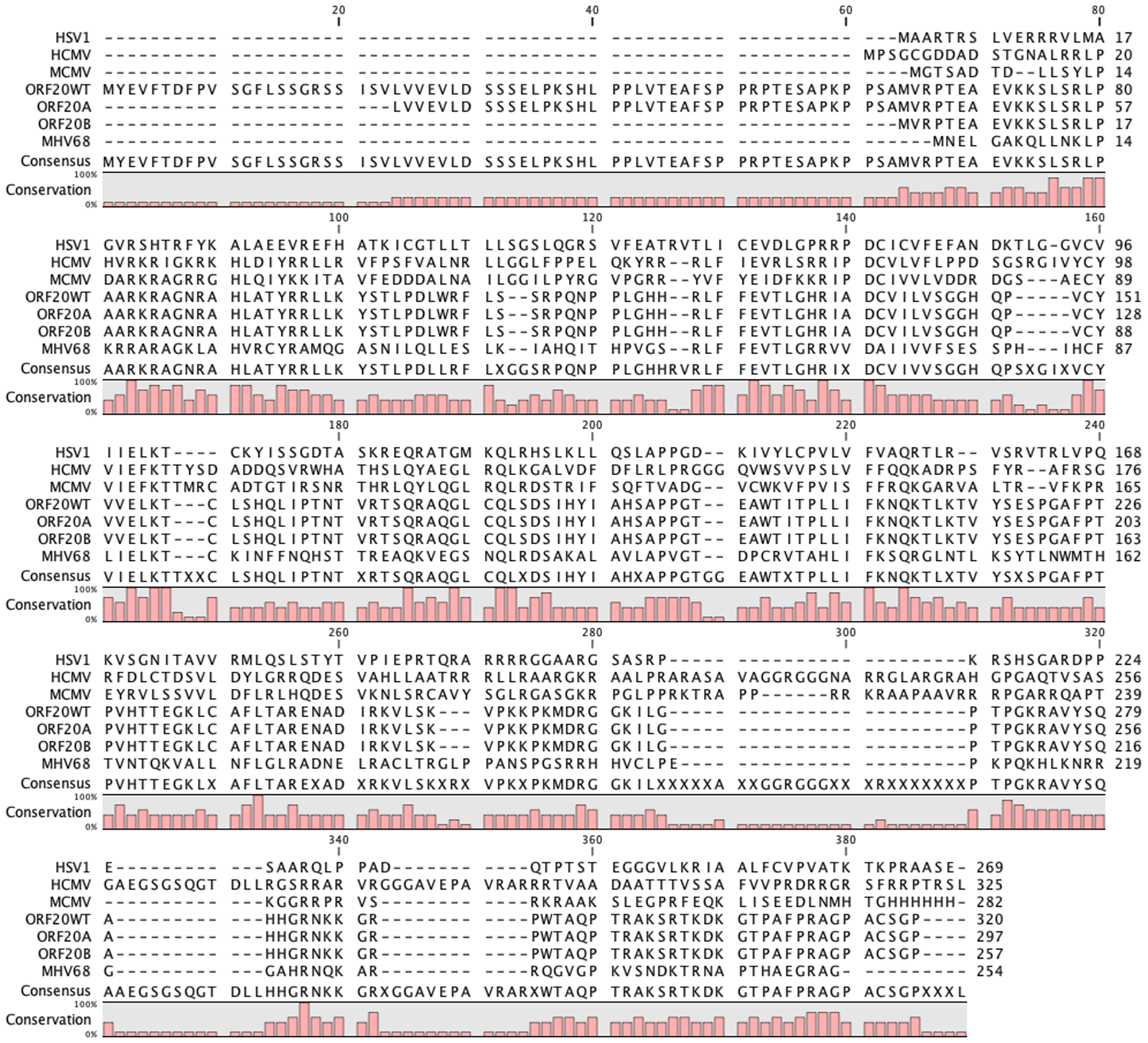

Supplement: S3 Fig — The amino acid sequences of HSV-1 UL24, HCMV UL76, MCMV M76, KSHV ORF20WT (FL with genomic ORF20A and ORF20B start codons), KSHV ORF20A, KSHV ORF20B, and MHV68 ORF20 were aligned using Clustal W2. (TIF) [file ppat.1006937.s003.tif]

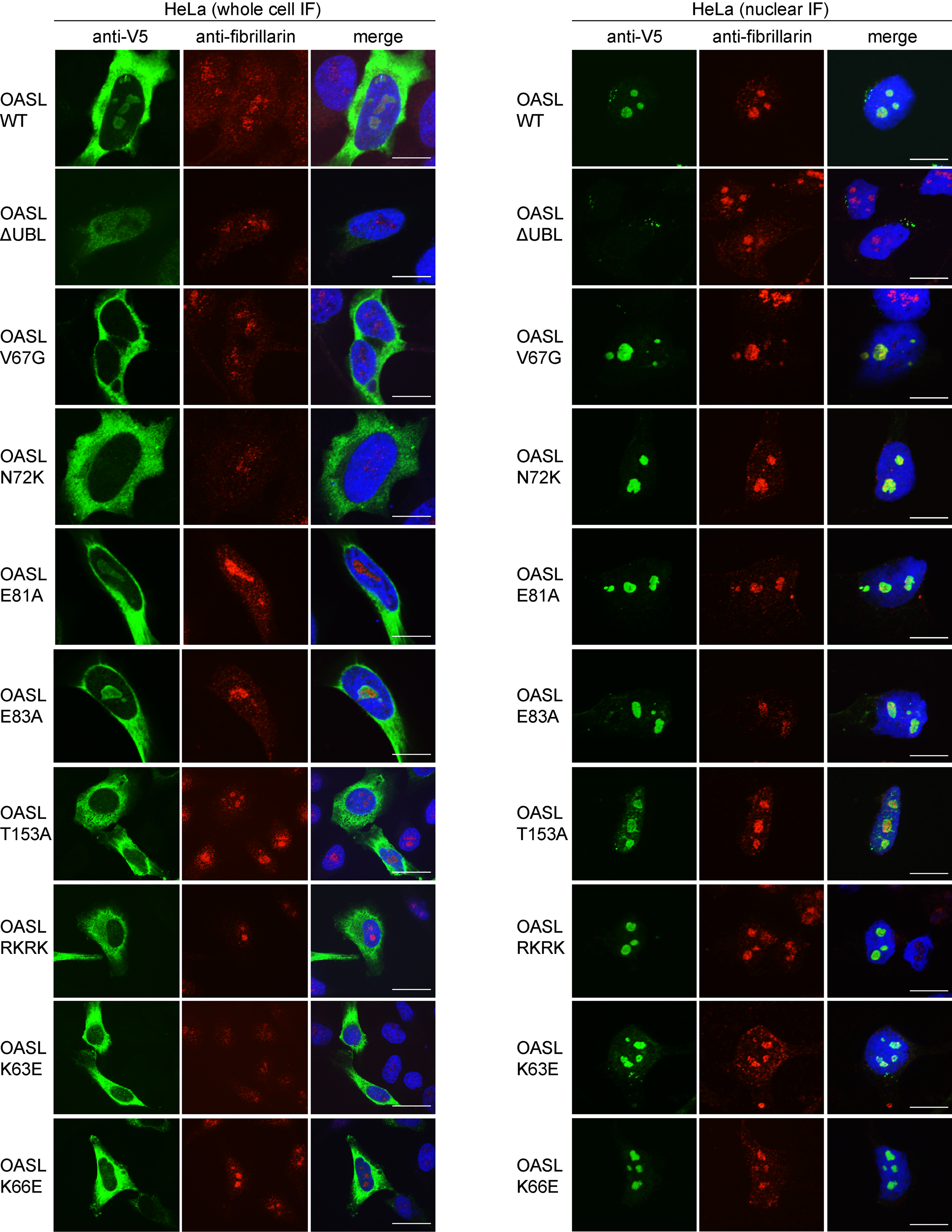

Supplement: S4 Fig — HeLa cells were transfected with the indicated plasmid and processed for whole cell and nuclear anti-V5 (green) and anti-fibrillarin (red) immunofluorescence. Nuclei were counterstained with Hoechst (blue). Images are representative of three independent experiments. Scale bar = 20 μm. (TIF) [file ppat.1006937.s004.tif]

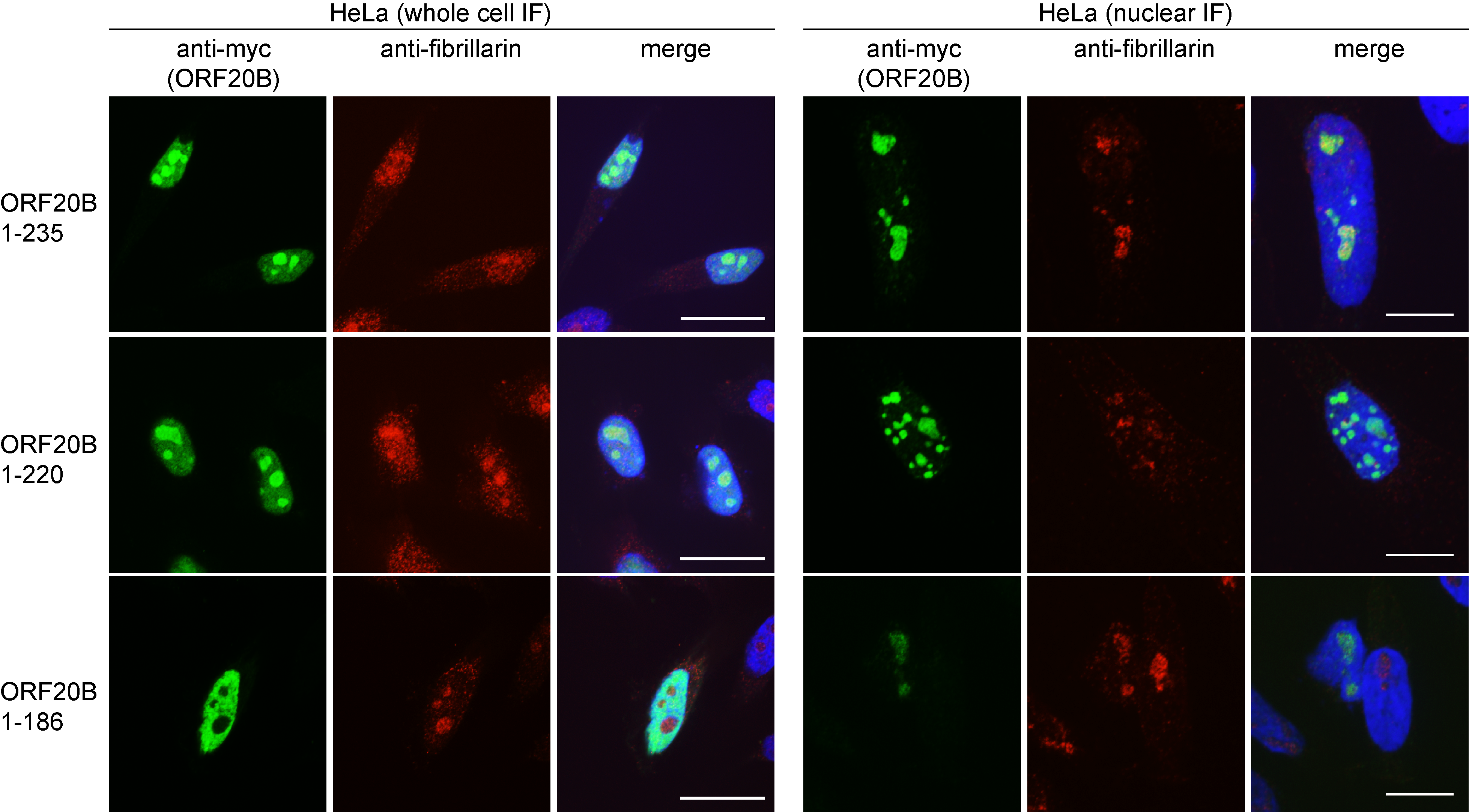

Supplement: S5 Fig — HeLa cells were transfected with plasmids expressing the indicated myc-tagged ORF20B deletion mutant plasmid and processed for whole cell and nuclear anti-myc (green) and anti-fibrillarin (red) immunofluorescence. Nuclei were counterstained with Hoechst (blue). Images are representative of three independent experiments. Scale bar = 30 μm (whole cell IF) and 15 μm (nuclear IF) (TIF) [file ppat.1006937.s005.tif]

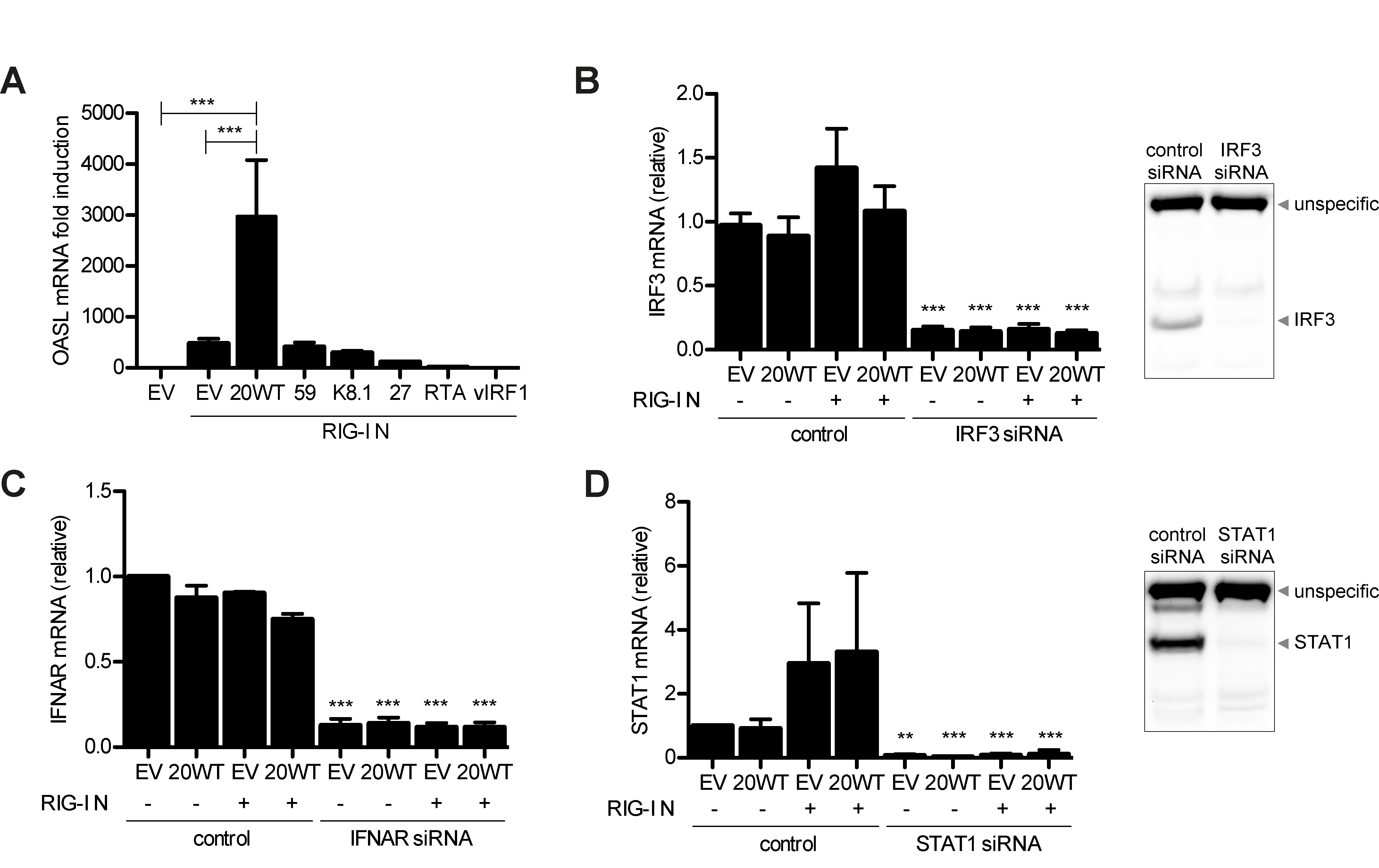

Supplement: S6 Fig — (A) 293T cells were co-transfected with the indicated plasmids for 24 h. The amount of OASL mRNA was determined by q-RT-PCR. (B, C, D) IRF3, IFNAR, or STAT1 mRNA levels were measured in the same samples described in Fig 9D. (A-D) Data shown are means + SD of duplicates from at least two experiments. Statistical significance was measured by one-way ANOVA followed by Tukey’s posttest ** P<0.01, *** P<0.001 (B, D) In parallel with preparation of samples for qPCR, protein lysates were prepared and analyzed for (B) IRF3 or (D) STAT1 expression by immunoblotting. (TIF) [file ppat.1006937.s006.tif]

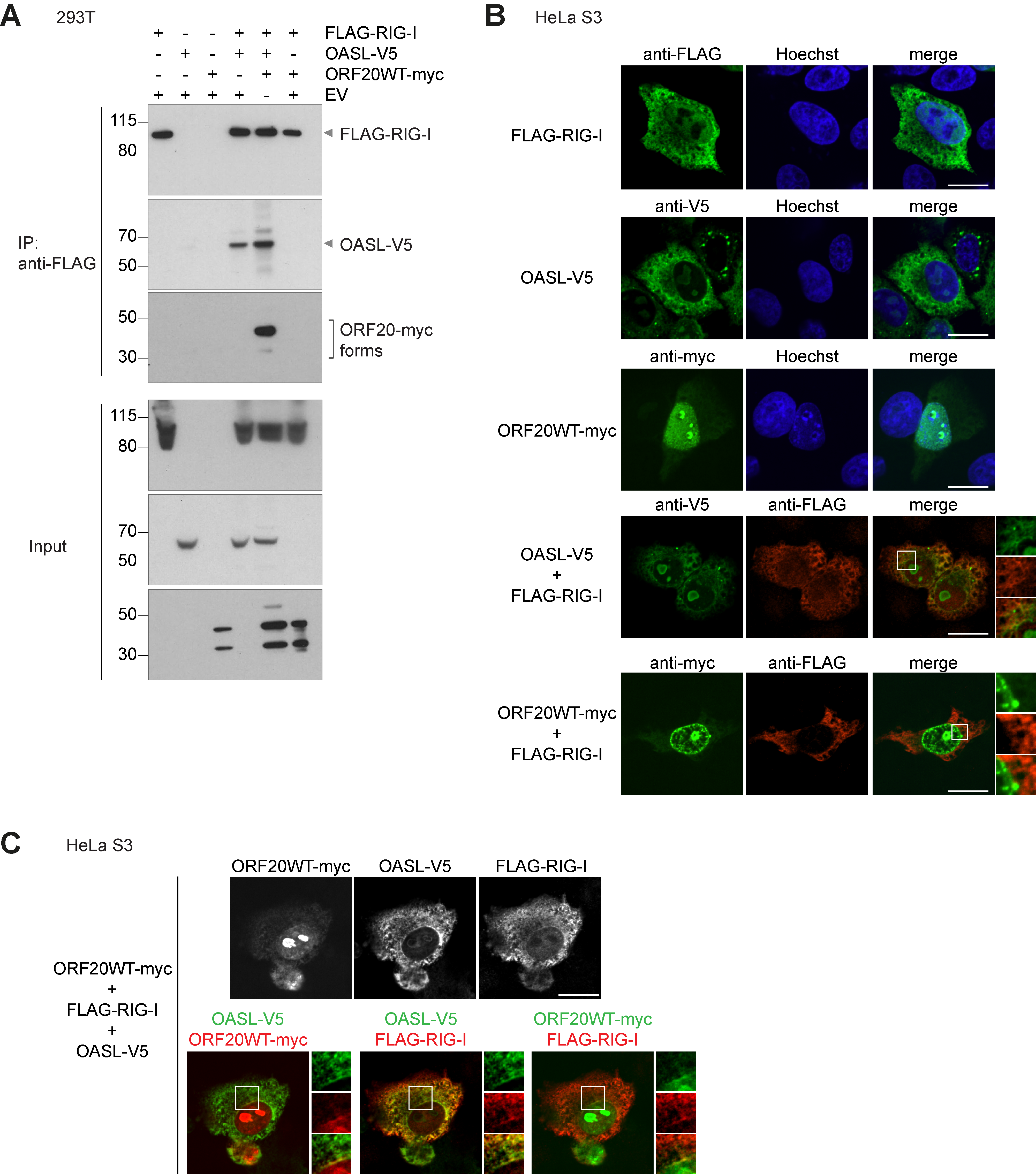

Supplement: S7 Fig — (A) 293T cells were transfected with the indicted combinations of FLAG-RIG-I, OASL-V5, ORF20WT-myc, and/or EV. NP40 lysates were subjected to anti-FLAG IP. Input lysates and immunoprecipitates were subjected to anti-FLAG, anti-V5, and anti-myc immunoblotting. (B and C) HeLa S3 cells on glass coverslips were transfected with the indicated plasmids, then processed for anti-FLAG, -V5, or -myc immunofluorescence as appropriate. Nuclei were counterstained with Hoechst. Scale bar = 20 μm. (TIF) [file ppat.1006937.s007.tif]
